# Demographics and regional trends of ischemic heart disease-related mortality in older adults in the United States, 1999–2020

**DOI:** 10.1371/journal.pone.0318073

**Published:** 2025-01-24

**Authors:** Fakhar Latif, Muhammad Moiz Nasir, Wajeeh Ur Rehman, Mohammed Hamza, Jishanth Mattumpuram, Komail Khalid Meer, Helme Silvet, Alon Yarkoni, Mouhamed Amr Sabouni, Nabil Braiteh, Keyoor Patel, Abdulqadir J. Nashwan

**Affiliations:** 1 Department of Internal Medicine, Dow University of Health Sciences, Karachi, Pakistan; 2 Department of Internal Medicine, United Health Services Hospital, Johnson City, NY, United States of America; 3 Department of Internal Medicine, Guthrie Medical Group, Cortland, NY, United States of America; 4 Division of Cardiology, Department of Medicine, University of Louisville School of Medicine, Louisville, KY, United States of America; 5 Department of Cardiology, Veterans Affairs, Loma Linda Healthcare System, Loma Linda, CA, United States of America; 6 UHS Heart & Vascular Institute, United Health Services Hospital, Johnson City, NY, United States of America; 7 Department of Cardiovascular Disease, University of Alabama at Birmingham, Birmingham, AL, United States of America; 8 Department of Cardiology, Mercy One Siouxland Heart and Vascular Center, Sioux City, IA, United States of America; 9 Nursing & Midwifery Research Department (NMRD), Hamad Medical Corporation, Doha, Qatar; 10 Department of Public Health, College of Health Sciences, QU Health, Qatar University, Doha, Qatar; Lithuanian University of Health Sciences, LITHUANIA

## Abstract

**Background:**

Ischemic heart disease (IHD) has a significant impact on public health and healthcare expenditures in the United States (US).

**Methods:**

We used data from the CDC WONDER database from 1999–2020 to identify trends in the IHD-related mortality of patients ≥ 75 years in the US. AAMRs per 100,000 population and APC were calculated and categorized by year, sex, race, and geographic divisions.

**Results:**

Between 1999 and 2020, a total of 8,124,568 IHD-related deaths were recorded. Notable declines in AAMR were observed from 1999 to 2014 (APC: -3.86) and from 2014 to 2018 (APC: -2.55), with an overall increase from 2018 to 2020 (APC: 3.76). Older men consistently demonstrated higher AAMRs than older females, with AAMRs for both sexes decreasing steadily from 1999 to 2018 and increasing in 2020. When stratified by race/ethnicity, Whites (1931.7) had the highest AAMR, followed by Blacks (1836.5), American Indians (1510.5), Hispanics (1464.4), and Asians (1093.6). Furthermore, nonmetropolitan areas (2015.2) showed greater AAMRs than metropolitan areas (1841.8). The ≥ 85-year group consistently exhibited higher IHD-related mortality rates compared to the 75–84 years group. In comparison, the older group [≥75 years] (1873.0) consistently exhibited higher IHD-related AAMRs than the younger group [<75 years] (64.0) throughout the study, showing a significant disparity. Chronic IHD (1552.0) consistently showed the highest AAMRs throughout the study, surpassing myocardial infarction (515.6), other ischemic heart diseases (24.0), and angina pectoris (5.6).

**Conclusion:**

Targeted interventions and resource allocation are crucial for areas with high IHD-related mortality. Public health policies should address demographic and geographical disparities, with further research for effective strategies.

## Introduction

Ischemic heart diseases (IHD) are a group of cardiac conditions that are marked by insufficient blood flow to the myocardium due to an obstruction in coronary arteries, causing an imbalance between the demand and supply of myocardial oxygen [1]. The primary coronary risk factors comprise hypertension, age, metabolic syndromes, genetics, and smoking, among many other causes [2]. Due to a variety of common risk factors, IHD contributes significantly to the global burden of disease.

As per the reports of World Health Organization (WHO), IHD accounted for 8.9 million deaths worldwide in 2019, representing about 16% of the total deaths globally [3]. Despite a reduction in its death rates over the years, the global burden of IHD has surged by 29% from 1990 to 2010, with the largest increase in mortality since 2000 [3, 4]. These alarming statistics are proof that IHD poses a significant threat to sustainable development in the 21^st^ century by causing disastrous increases in US health expenditures. As such, costs associated with IHD healthcare are expected to soar by 41%, increasing from $126.2 billion in 2010 to 177.5 billion in 2040 [4]. Furthermore, heart failure, which is the most prevalent consequence of IHD, results in an estimated annual global cost of $108 billion [5]. Although the recent statistics are very ominous, it is important to acknowledge the fact that there has been substantial improvement in hospitalization and readmission rates from IHD in the US, owing to recent advances in technology and medical therapy [6].

Through this study, we aim to analyze the IHD-related mortality trends in the United States of America (USA) over 20 years with regard to demographical and geographical variations. The analysis will underscore the importance of targeted interventions and resource allocation to address areas with higher mortality rates and shed light on health inequities among the elderly. Additionally, our study will also be able to highlight the impact of policy decisions and public health interventions aimed at improving cardiovascular health outcomes. In essence, the study aims to contribute to understanding IHD-related mortality trends, providing a foundation for further studies and informing strategies to reduce mortality rates and improve public health outcomes related to cardiovascular disease.

## Methods

### Data source

The analysis was based on the data sourced from the Centers for Disease Control and Prevention (CDC) Wide-ranging Online Data for Epidemiologic Research (WONDER) database, which includes mortality causes from death certificates from 50 states and the District of Columbia in the United States [7]. The underlying cause of death (UCD) refers to the disease directly leading to the demise, while multiple cause of death (MCD) encompass other conditions associated with the fatal outcome. Both UCD and MCD are classified using the International Classification of Diseases (Tenth Edition).We analyzed Multiple Cause-of-Death (MCD) Public Use record death certificates to identify deaths related to ischemic heart diseases in individuals of age greater than 75 years. This study included all decedents with IHD (I20.0, I20.1, I20.8, I20.9, I21.0, I21.1, I21.2, I21.3, I21.4, I21.9, I22.0, I22.1, I22.8, I22.9, I24.1, I24.8, I24.9, I25.0, I25.1, I25.2, I25.3, I25.4, I25.5, I25.6, I25.8, I21.9) as MCD [8]. Therefore, this study included data from the death certificates that either identified IHD as the underlying cause of death or as one of the contributing causes of death.

The study did not necessitate institutional review board (IRB) approval because it exclusively analyzed government-issued public use data, devoid of individually identifiable information. Furthermore, the study was conducted in adherence to the STROBE (Strengthening the Reporting of Observational Studies in Epidemiology) guidelines [9].

### Data extraction

The number of IHD-related deaths and population size were extracted from 1999 to 2020. The data on age, sex, race and ethnicity, region, state, and place of death was also obtained. The patients aged ≥75 years were selected and divided into 10-year age groups. For race, patients were stratified into Hispanics, Non-Hispanic (NH) White, NH Black, NH Asian/Pacific Islander, and NH American Indian/Alaskan Native. Regions were categorized into Northeast, Midwest, South, and West, following the Census Bureau-defined regional divisions. The population was additionally classified into metropolitan, non-metropolitan, and rural counties in accordance with the National Center for Health Statistics Urban-Rural Classification Scheme [10].

### Statistical analysis

Crude mortality rates (CMRs) and age-adjusted mortality rates (AAMRs) per 100,000 population were calculated for IHD-related deaths. CMRs were derived by dividing the number of IHD-related deaths by the corresponding US population for that specific year. AAMRs were determined by standardizing the IHD-related deaths to the year 2000 US population [11]. Trends in both crude and age-adjusted mortality rates were analyzed using the Joinpoint Regression Program (Version 4.9.0.0, National Cancer Institute), which models consecutive linear segments on a log scale connected by joinpoints, where the segments converge [12]. Joinpoint regression has been extensively applied across various domains to assess trends in time series data, such as cancer mortality[13]. A notable advantage of this method is its ability to identify points where significant changes in trends occur, allowing for a more detailed analysis of shifts within longitudinal data. The annual percent change (APC) with its 95% CI was also calculated. Slopes were considered increasing or decreasing if the estimated slope differed significantly from zero. The statistical significance was determined by 2-sided t-testing (p = 0.05).

## Results

Between 1999 and 2020, a total of 8,124,568 IHD-related deaths were recorded among adults aged 75 years or older [**S1 Table**]. Information regarding the location of death was available for 7,803,875 of these deaths. Among these, 41% occurred in medical facilities, 30% in nursing homes or long-term care facilities, 26% at home, and 3% in hospices. [**S2 Table**]

### Annual trend for IHD-related AAMR

**Fig 1:**
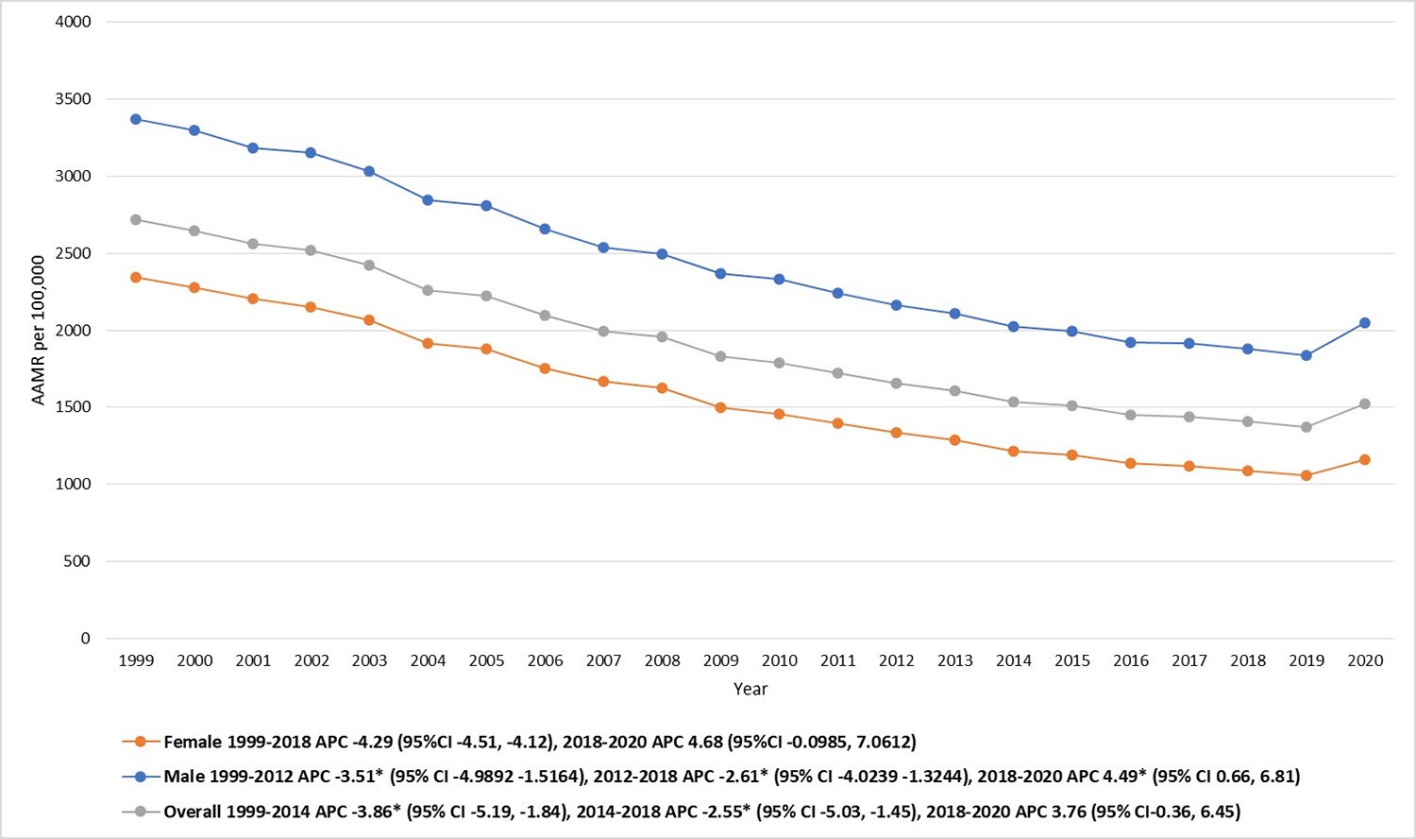
Ischemic heart disease-related mortality trends in older adult population and stratified for gender in 1999–2020.

The AAMRs for IHD-related mortality in the elderly has gradually declined from 2718.1 in 1999 to 1521.5 in 2020. Additionally, a noticeable decrease in AAMR was seen from 1999 to 2014 (APC: -3.86; 95% CI: -5.2 to -1.8). Subsequently, the AAMRs showed a significant decline from 2014–2018 (APC: -2.55; 95% CI: -5.03 to -1.45), which was followed by an overall increment of AAMR from 2018 to 2020 (APC: 3.76; 95% CI: -0.3 to 6.45). [**Fig 1, Table 1 and S3 Table**]

**Table 1:** Deaths, overall AAMRs, and Annual Percent Change (APC) of ischemic heart diseases-related mortality in older adults in the United States, 1999 to 2020.

Variable	Deaths (n)	AAMR (95% CI)	Trend Segment	Year Interval	APC (95% CI)	AAPC (95% CI)
**Overall**	8,124,568	1873.0 (1871.7, 1874.3)	1	**1999–2014**	-3.86* (-5.19, -1.84)	-2.91* (-3.12, -2.72)
			2	**2014–2018**	-2.55* (-5.03, -1.45)	
			3	**2018–2020**	3.76 (-0.36, 6.45)	
**Sexual Disparities**						
**Men**	3,844,256	2391.0 (2388.7, 2393.4)	1	**1999–2012**	-3.51* (-4.99, -1.52)	-2.51* (-2.78, -2.31)
			2	**2012–2018**	-2.61* (-4.02, -1.32)	
			3	**2018–2020**	4.49* (0.66, 6.81)	
**Women**	4,280,312	1547.6 (1546.1, 1549.1)	1	**1999–2018**	-4.29 (-4.51, -4.12)	-3.46* (-3.79, -3.28)
			2	**2018–2020**	4.68 (-0.10, 7.06)	
**Racial Disparities**						
**White**	6,944,019	1931.7 (1930.2, 1933.1)	1	**1999–2012**	-3.77* (-4.83, -3.01)	-2.84* (-3.1, -2.69)
			2	**2012–2018**	-2.64* (-3.79, -1.41)	
			3	**2018–2020**	3.24 (-0.40, 5.41)	
**Black or African American**	604,104	1836.5	1	**1999–2002**	-0.85 (-2.89, 3.58)	-2.79* (-3.12, -2.47)
			2	**2002–2011**	-4.83* (-6.83, -4.41)	
			3	**2011–2018**	-3.66* (-5.47, -2.00)	
			4	**2018–2020**	7.87* (3.27, 10.53)	
**American Indian or Alaska Native**	25,582	1510.5 (1492.0, 1529.1)	1	**1999–2020**	-2.59* (-3.01, -2.13)	-3.18* (-3.58, -2.75)
**Hispanic or Latino**	378,557	1464.4 (1459.7, 1469.1)	1	**1999–2018**	-4.37* (-4.71, -4.05)	-2.87* (-3.29, -2.59)
			2	**2018–2020**	12.52* (6.06, 16.08)	
**Asian or Pacific Islander**	153,435	1093.6 (1088.1, 1099.1)	1	**1999–2018**	-3.98* (-4.25, -3.72)	-2.95* (-3.26, -2.71)
			2	**2018–2020**	7.38* (2.37, 10.14)	
**Age Disparities**						
**75–84 Years**	3,718,866	1245.8 (1244.6, 1247.1)	1	**1999–2012**	-4.08* (-5.00, -3.84)	-3.00* (-3.23, -2.86)
			2	**2012–2018**	-2.99* (-3.96, -1.66)	
			3	**2018–2020**	4.35* (1.05, 6.48)	
**85+ Years**	4,405,702	3686.4 (3682.9, 3689.8)	1	**1999–2017**	-3.64* (-3.87, -3.46)	-2.86* (-3.10,-2.67)
			2	**2017–2020**	1.95 (-0.60, 6.19)	
**Younger Adults vs. Older Adults**						
**Younger than 75 Years**	4,673,049	64.0 (63.9, 64.0)	1	**1999–2010**	-3.91* (-4.60, -3.51)	-2.07* (-2.32, -1.89)
			2	**2010–2018**	-1.36* (-2.30, -0.54)	
			3	**2018–2020**	5.55* (1.78, 7.47)	
**Older than 75 Years**	8,124,568	1873.0 (1871.7, 1874.3)	1	**1999–2014**	-3.86* (-5.19, -1.84)	-2.91* (-3.12, -2.72)
			2	**2014–2018**	-2.55* (-5.03, -1.45)	
			3	**2018–2020**	3.76 (-0.36, 6.45)	
**Rural/Urban Disparities**						
**Nonmetropolitan areas**	1,566,865	2015.2 (2012.0, 2018.4)	1	**1999–2018**	-3.00* (-3.29, -2.81)	-2.23* (-2.65, -2.04)
			2	**2018–2020**	5.33 (-0.44, 7.73)	
**Metropolitan area**	6,557,703	1841.8 (1840.4, 1843.2)	1	**1999–2014**	-4.02* (-5.25, -2.14)	-3.06* (-3.31, -2.87)
			2	**2014–2018**	-2.64* (-4.88, -1.55)	
			3	**2018–2020**	3.77 (-0.21, 6.42)	
**Regional Disparities**						
**North-eastern Census Region**	1,876,745	2079.9 (2076.9, 2082.9)	1	**1999–2018**	-3.86* (-4.09, -3.70)	-3.11* (-3.45, -2.92)
			2	**2018–2020**	4.41 (-0.59, 6.74)	
**Mid-western Census Region**	1,935,508	1923.1 (1920.4, 1925.8)	1	**1999–2010**	-3.68* (-4.95, -3.38)	-2.59* (-2.82, -2.44)
			2	**2010–2018**	-2.88* (-3.47, -1.74)	
			3	**2018–2020**	4.87* (1.55, 6.91)	
**Southern Census Region**	2,764,844	1815.1 (1813.0, 1817.3)	1	**1999–2002**	-2.46 (-3.76, 0.30)	-2.72* (-2.95, -2.50)
			2	**2002–2011**	-4.37* (-6.06, -4.03)	
			3	**2011–2018**	-2.79* (-3.67, -1.56)	
			4	**2018–2020**	4.91* (1.71, 6.97)	
**Western Census Region**	1,547,471	1706.0 (1703.3, 1708.7)	1	**1999–2003**	-2.80* (-3.63, -0.73)	-2.95* (-3.15, -2.78)
			2	**2003–2009**	-4.74* (-6.20, -4.16)	
			3	**2009–2018**	-3.03* (-3.58, -2.39)	
			4	**2018–2020**	2.66 (-0.23, 4.29)	
**Causes**						
**Angina Pectoris**	24,618	5.6 (5.6, 5.7)	1	**1999–2008**	-13.82* (-16.33,-11.57)	-3.65* (-4.47,-3.03)
			2	**2008–2014**	-4.26 (-16.71, 0.15)	
			3	**2014–2017**	21.58 (-5.09, 27.58)	
			4	**2017–2020**	8.07 (-3.21, 14.65)	
**Chronic Ischemic Heart Diseases**	6,739,489	1552.0 (1550.8, 1553.2)	1	**1999–2014**	-3.68* (-4.82, -2.05)	-2.68* (-2.92,-2.52)
			2	**2014–2018**	-2.46* (-4.26, -1.40)	
			3	**2018–2020**	4.62* (1.00, 7.05)	
**Myocardial Infarction**	2,224,233	515.6 (515.0, 516.3)	1	**1999–2002**	-3.90* (-5.11, -1.99)	-4.56* (-4.76, -4.40)
			2	**2002–2009**	-6.54* (-7.79, -6.13)	
			3	**2009–2018**	-4.19* (-5.01, -3.75)	
			4	**2018–2020**	-0.11 (-3.12, 1.70)	
**Other Ischemic Heart Diseases**	103,985	24.0 (23.9, 24.2)	1	**1999–2004**	-4.15* (-10.59, -1.46)	-0.97* (-1.46,-0.48)
			2	**2004–2007**	9.39* (2.94, 12.92)	
			3	**2007–2020**	-1.99* (-2.85, -1.36)	

### IHD-related AAMR stratified by sex

Throughout the study duration, older men consistently demonstrated higher age-adjusted mortality rates (AAMRs) than older females, despite both groups showing a downward trend (overall AAMR Males: 2391.0 [95% CI: 2388.7–2393.4]; Females: 1547.6 [95% CI: 1546.1–1549.1]) **[Table 1]**. The AAMR for males decreased from 3371.0 in 1999 to 2051.2 in 2020, while the AAMR for females reduced from 2346.0 in 1999 to 1157.6 in 2020.

The AAMRs for males between 1999 and 2012 showed a decreasing trend [APC: -3.51 (95% CI -4.9892, -1.5164)]. From 2012 to 2018, the AAMRs continued to decrease, albeit at a slower rate [APC: -2.61 (95% CI -4.0239, -1.3244)]. However, a subsequent increase in AAMRs was observed till 2020, [APC: 4.49 (95% CI 0.66, 6.81)]. [**Fig 1, Table 1 and S4 Table**]

For females, a similar pattern was observed. The AAMRs decreased steadily from 1999 to 2018 [APC of -4.29 (95% CI -4.51, -4.12)], followed by a moderate increase till 2020 [APC: 4.68 (95% CI -0.0985, 7.0612)]. [**Fig 1, Table 1 and S4 Table**]

### IHD-related AAMR stratified by race

**Fig 2:**
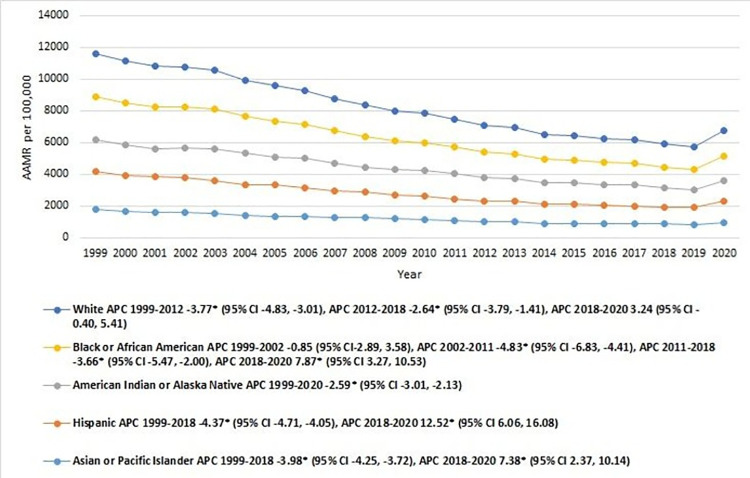
Ischemic heart disease-related mortality trends in older adult population stratified for differential racial groups in 1999–2020.

When stratified by race/ethnicity, NH Whites had the highest AAMR, followed by NH Black, NH American Indians, Hispanics, and Asian or Pacific Islander (overall AAMR NH White: 1931.7 [95% CI: 1930.2–1933.1]; NH Black or African American: 1836.5 [95% CI: 1831.9–1841.2]; NH American Indian or Alaska Native: 1510.5 [95% CI: 1492.0–1529.1]; Hispanic or Latino: 1464.4 [95% CI: 1459.7–1469.1]; NH Asian or Pacific Islander: 1093.6 [95% CI: 1088.1–1099.1]) **[Table 1]**. Asian/Pacific Islander and Hispanic/Latino showed a gradual decline in mortality rate from 1999 till 2018 [APC: Asian or Pacific Islanders: -3.98* (95% CI -4.25, -3.72); Hispanic/Latino: -4.37* (95% CI -4.71, -4.05)], followed by a much sharper rise for till 2020 [APC: Asian or Pacific Islander: 7.38* (95% CI 2.37, 10.14); Hispanic/Latino: 12.52* (95% CI 6.06, 16.08)]. Conversely, NH Whites showed a slight decline from 1999–2012 [APC: -3.77* (95% CI -4.83, -3.01)], which was followed by another significant decline till 2018 [APC: -2.64* (95% CI -3.79, -1.41)]. Subsequently, a sharp rise for was seen in the AAMRs till 2020 [APC: 3.24 (95% CI -0.40, 5.41)]. American Indian/ Alaska Native remain an exception, showing a more gradual but continuous decline throughout the study period [APC: -2.59 (95% C.I: -3.01, -2.13)]. Significant variations in AAMR were observed for NH Black/African American, showing a steady rate from 1999 to 2002 (APC: -0.85; 95% C.I: -2.89, 3.58), followed by a sharper decline till 2011 [APC: -4.83 (95% C.I: -6.83, 4.41)], a more gradual decline till 2018 [APC: -3.66 (95% C.I: -5.47, -2.00)], and finally a sharp rise till 2020 [APC: 7.87 (95% C.I: 3.27, 10.53)] [**Fig 2, Table 1 and S5 Table**].

### IHD-related AAMR stratified by age

**Fig 3:**
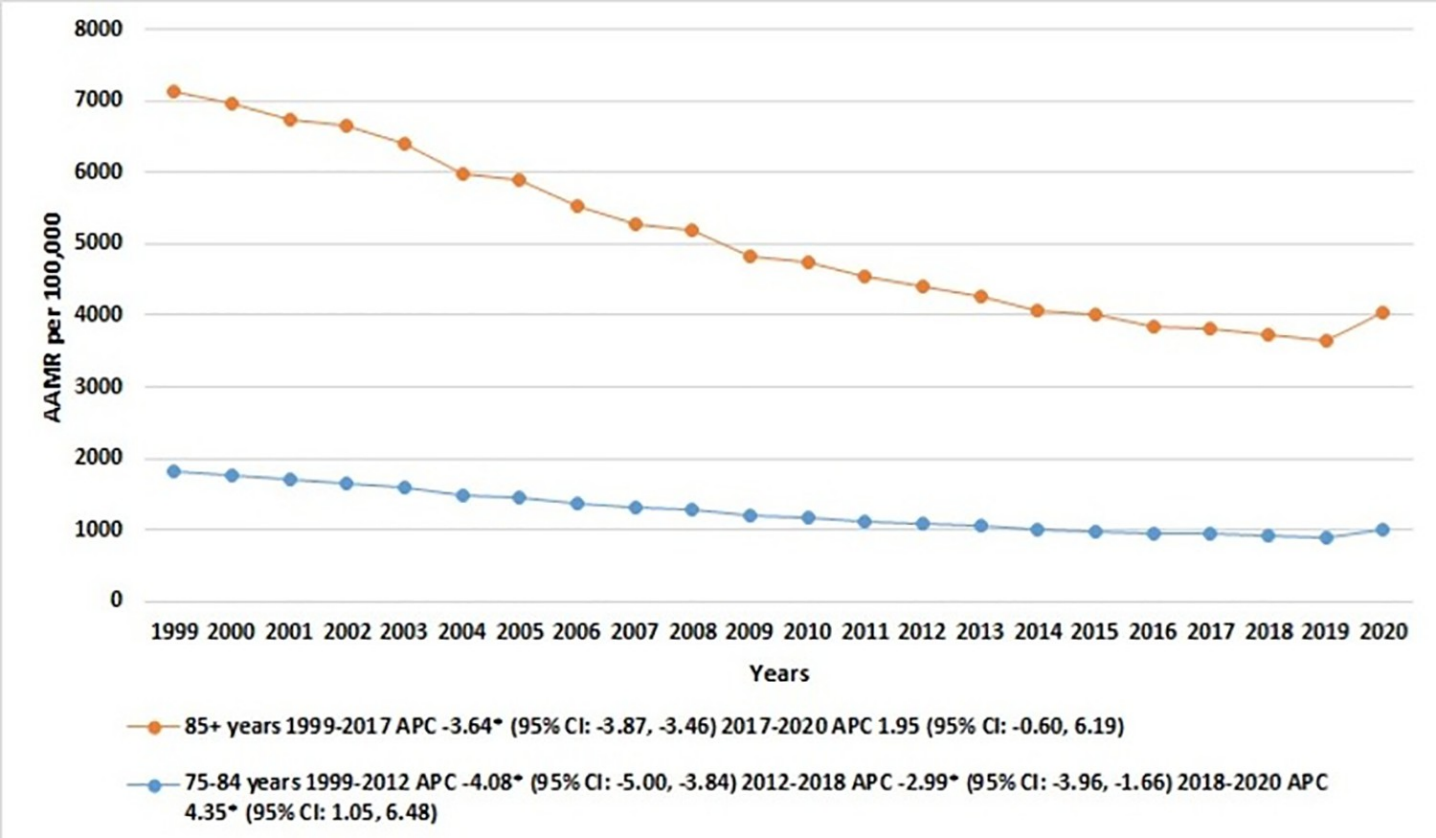
Ischemic heart disease-related mortality trends in older adult population and stratified for age groups in 1999–2020.

When stratifying the older adults into two ten-year age brackets, the ≥85 years age group consistently displayed higher IHD-related mortality rates compared to the 75–84 years age group. In patients aged ≥85 years, the mortality rates steadily fell from 1999 to 2017 [APC: -3.64* (95% CI: -3.87, -3.46)], followed by a minimal increase from 2017 to 2020 [APC: 1.95 (95% CI: -0.60, 6.19)]. Meanwhile, in the 75–84 years group, the mortality rates fell sharply between 1999 and 2012 [APC: -4.08* (95% CI: -5.00, -3.84)], then remained relatively stable between 2012 and 2018 [APC: -2.99* (95% CI: -3.96, -1.66)], before rising significantly from 2018 to 2020 [APC: 4.35* (95% CI: 1.05, 6.48)]. [**Fig 3, Table 1 and S6 Table**].

**Fig 4:**
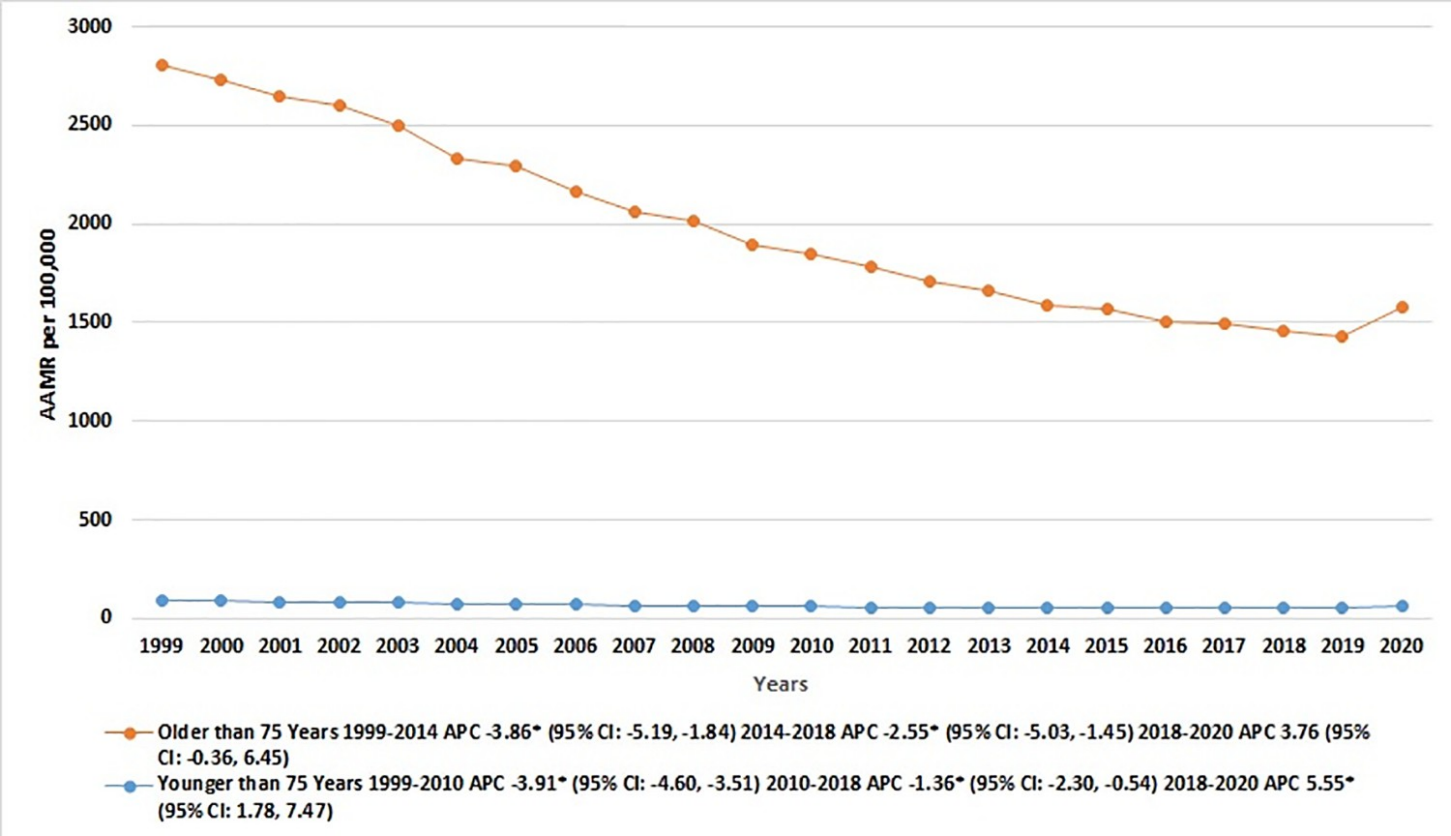
Ischemic heart disease-related mortality trends in older adult population and stratified for older vs. young population in 1999–2020.

When comparing older adults (patients aged ≥75 years) to younger individuals (patients aged <75 years), it was evident that the older group invariably exhibited higher IHD-related AAMRs throughout the study period, with a notable disparity between the two groups (overall AAMR Age ≥75 years: 1873 [95% CI: 1871.1–1874.3]; Age <75 years: 64 [95% CI: 63.9–64]). For patients aged ≥75 years, there was a marked reduction in the AAMR from 1999 to 2014 [APC: -3.86* (95% CI: -5.19, -1.84)], followed by a more gradual decline from 2014 to 2018 [APC: -2.55* (95% CI: -5.03, -1.45)], and a subsequent rise from 2018 to 2020 [APC: 3.76 (95% CI: -0.36, 6.45)]. In contrast, the AAMR for those aged <75 years experienced a steeper decrease from 1999 to 2010 [APC: -3.91* (95% CI: -4.60, -3.51)], a less marked reduction from 2010 to 2018 [APC: -1.36 (95% CI: -2.30, -0.54)], and a noticeable uptick from 2018 and 2020 [APC: 5.55* (95% CI: 1.78, 7.47)]. [**Fig 4, Table 1 and S7 Table**].

### IHD-related AAMR stratified by geography

#### Rural-urban classification

**Fig 5:**
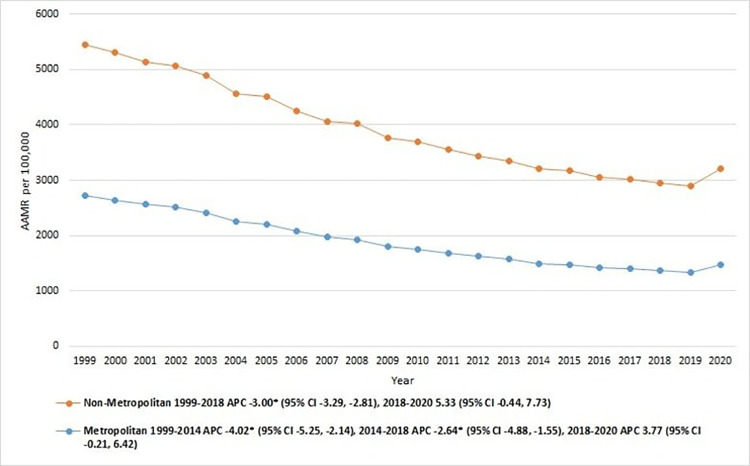
Ischemic heart disease-related mortality trends in older adult population and stratified for metropolitan vs. non-metropolitan in 1999–2020.

Similarly, nonmetropolitan areas showed greater AAMRs for mortality due to IHD than metropolitan areas, with overall AAMRs of 2015.2 (95% CI: 2012–2018.4) and 1841.8 (95% CI 1840.4–1843.2), respectively **[Table 1]**. Furthermore, the AAMRs of nonmetropolitan areas significantly decreased from 1999 to 2018 [APC: -3.00 (95% CI -3.29 to -2.81)] while subsequently increasing from 2018 to 2020 [APC: 5.33 (95% CI -0.44, 7.73)]. Metropolitan areas demonstrated a substantial decline from 1999 to 2014 [APC: -4.02 (95% CI -5.25 to -2.14)]. This was followed by a period of another significant reduction until 2018 [APC: -2.64 (95% CI -4.88 to -1.55)]. AAMRs remained stable from 2018 to 2020 [APC: 3.77 (95% CI -0.21, 6.42)]. [**Fig 5, Table 1 and S8 Table**].

#### Census regions

**Fig 6:**
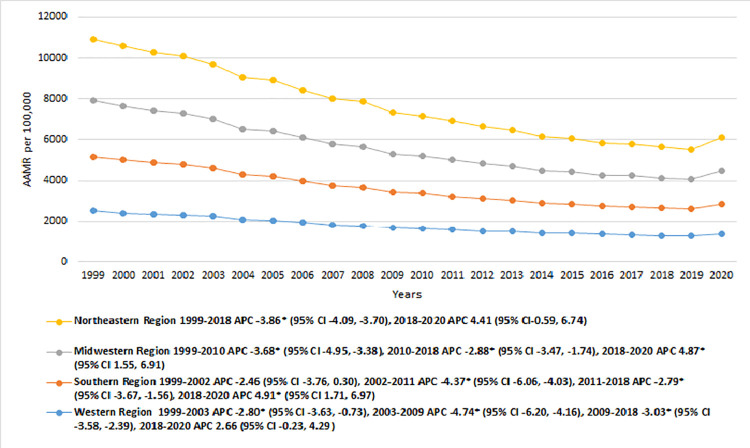
Ischemic heart disease-related mortality trends in older adult population and stratified for census regions in 1999–2020.

Among the four census regions, the Northeastern region consistently exhibited the highest AAMRs throughout the study duration, followed sequentially by the Midwestern, Southern, and Western regions (overall AAMR Northeast: 2079.9 [95% CI: 2076.9–2082.9]; Midwest: 1923.1 [95% CI: 1920.4–1925.8]; South: 1815.1 [95% CI: 1813–1817.3]; West: 1706 [95% CI: 1703.3–1708.7]). A similar trend of IHD-related AAMRs was observed across the four census regions, with a marked decline from 1999 to 2018, followed by a significant increase between 2018 and 2020. In the Northeastern region, the AAMR substantially decreased from 1999 to 2018 [APC: -3.86* (95% CI: -4.09, -3.70)], before experiencing a prominent rise from 2018 to 2020 [APC: 4.41 (95% CI: -0.59, 6.74)]. Meanwhile, the AAMR for the Midwest steeply reduced from 1999 to 2010 [APC: -3.68* (95% CI: -4.95, -3.38)], followed by a less dramatic reduction from 2010 to 2018 [APC: -2.88* (95% CI: -3.47, -1.74)], and subsequently, a drastic escalation from 2018 to 2020 [APC: 4.87* (95% CI: 1.55, 6.91)]. Similarly, the Southern and Western regions also demonstrated a downward trend of IHD-related AAMRs at a variable pace between 1999 and 2018, before displaying a pronounced increase from 2018 to 2020 [APC South: 4.91* (95% CI: 1.17, 6.97); West: 2.66 (95% CI: -0.23, 4.29)]. [**Fig 6, Table 1 and S9 Table**].

### IHD-related AAMR stratified by causes

When examining the different causes of IHD, chronic ischemic heart disease consistently demonstrated the highest AAMRs throughout the study period, significantly outpacing myocardial infarction, other ischemic heart diseases, and angina pectoris (overall AAMR Chronic IHD: 1552 [95% CI: 1550.8–1553.2]; Myocardial Infarction: 515.6 [95% CI: 515–516.3]; Other IHD: 24 [95% CI: 23.9–24.2]; Angina Pectoris: 5.6 [95% CI: 5.6–5.7]).

**Fig 7:**
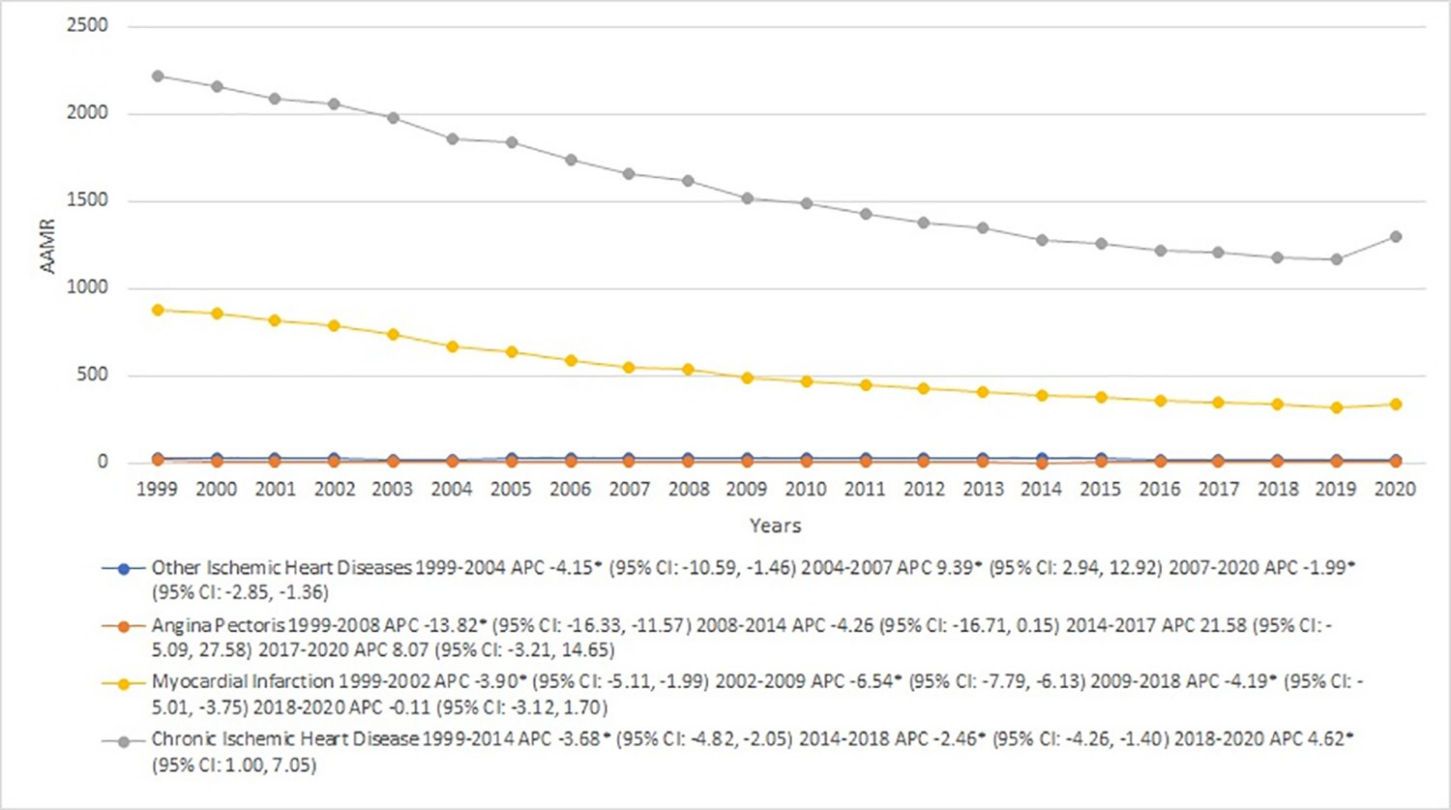
Ischemic heart disease-related mortality trends in older adult population and stratified for causes in 1999–2020.

For chronic IHD, the AAMR steadily diminished from 1999 to 2014 [APC: -3.68* (95% CI: -4.82, -2.05)], continued to decrease at a slower pace between 2014 and 2018 [APC: -2.46* (95% CI: -4.26, -1.40)], and then experienced a notable surge from 2018 to 2020 [APC: 4.62* (95% CI: 1.00, 7.05)]. The AAMR for myocardial infarction showed a slight reduction from 1999 to 2002 [APC: -3.90* (95% CI: -5.11, -1.99)], followed by a steep drop from 2002 to 2009 [APC: -6.54* (95% CI: -7.79, -6.13)]. This decreasing trend continued from 2009 to 2018 [APC: -4.19* (95% CI: -5.01, -3.75)], before stabilizing between 2018 and 2020 [APC: -0.11 (95% CI: -3.12, 1.70)]. Other ischemic heart diseases displayed a unique trend, with a marked reduction in AAMR from 1999 to 2004 [APC: -4.15* (95% CI: -10.59, -1.46)], a significant rise from 2004 to 2007 [APC: 9.39* (95% CI: 2.94, 12.92)], and a relatively stable period from 2007 to 2020 [APC: -1.99* (95% CI: -2.85, -1.36)]. For angina pectoris, the AAMR drastically fell from 1999 to 2008 [APC: -13.82* (95% CI: -16.33, -11.57)], followed by a slower decline from 2008 to 2014 [APC: -4.26 (95% CI: -16.71, 0.15)]. Afterward, there was a sharp escalation from 2014 to 2017 [APC: 21.58 (95% CI: -5.09, 27.58)], and a more moderate rise from 2017 to 2020 [APC: 8.07 (95% CI: -3.21, 14.65)]. [**Fig 7, Table 1 and S10 Table**].

## Discussion

Using the deidentified national death certificate data from the CDC Wonder database we have identified several key findings of interest. First, we observed an overall decline in mortality from IHD in older adults in the US over the past 20+ years. Although this finding was true for either sex, males showed consistently higher mortality rates compared to females. Second, NH White older adults showed the highest IHD-related AAMR followed by NH Blacks, whereas Asians or Pacific Islanders showed the lowest and least variant AAMR compared to all other racial groups. Third, significant geographical variations were also observed, with rural areas showing the highest mortality rates. These results could prove impactful for efficient public health policy structuring.

IHD-related mortality has shown a gradual decline from 1999 onwards, with a recent rise seen from 2019 to 2020. This coincides with the COVID-19 pandemic period, where patients with pre-existing cardiovascular disease (CVD) risk factors showed a tendency towards worse outcomes and higher need for emergency cardiovascular care. Furthermore, data from the CDC dataset suggests that 20–35% of COIVD-19 associated mortality occurred in patients with pre-existing CVD and risk factors [14]. Studies suggest endothelial dysfunction, coagulopathy, and active inflammation to be aggravating factors for CVD, similar to diseases like diabetes and hypertension [15]. However, it is imperative to understand that the pre-pandemic trend depicting a decline in IHD-related mortality may be masked by a recent surge in mortality rates owing to the long-term effects of COVID-19 on the cardiovascular system.

Our analysis indicated that both men and women demonstrated a steady decline in IHD-related mortality rates, with women showing a slightly sharper decrease than men. Previous studies highlight the prominent gender-based disparities in diagnostic assessment of patients with IHD, where women remain evidently underdiagnosed, prone to prehospital delays in symptom presentation, present with higher rates of debilitating comorbidities including diabetes, and a consequent delay in aggressive treatment [16–18]. Data from the National Cardiovascular Data Registry (NCDR) suggested that delays in contact-to-reperfusion time resulted in higher rates of mortality in women compared to men [19]. Some studies indicate the dominance of male cardiologists in hospital settings to be a major contributing factor towards low patient satisfaction among female patients [20, 21]. While there remain several interconnected factors underlying the disparities, recent data hints at slight improvements in outcomes for female IHD patients. For instance, improved physician training based on uniform evidence-based protocols has improved long-term mortality rates in women receiving emergency ST-elevation myocardial infarction (STEMI) treatment [22]. Despite extensive research into the biological, pathophysiological, sex, gender, and socioeconomic-related determinants of IHD progression and presentation among either gender, practical implementations of this evident knowledge remain insufficient [23, 24].

We observed that both the metropolitan and nonmetropolitan areas showed a steady decline in the burden of IHD-related mortality, with the rate of decline being slightly higher in metropolitan areas [25]. Furthermore, delays in the transportation of patients with STEMI from the point of contact with primary emergency services to balloon inflation in the nearest primary percutaneous coronary intervention (PCI) facility may contribute to the urban/rural disparities. A study found that patients from rural regions had to travel 30 km further than those from urban areas to their nearest primary PCI facilities, thus leading to system delays [26]. Pre-hospital diagnosis using an electrocardiogram (ECG) will enable the EMS to bypass non-invasive medical facilities and limit the time delays. However, IHD-related AAMR remains consistently higher in nonmetropolitan areas compared to metropolitan areas, a disparity congruent with those observed in heart failure patients [27, 28]. This trend could be attributed to the relative inadequacy of advanced healthcare facilities and dedicated cardiac health centers, worsened by the low number of primary healthcare physicians and specialist physicians, such as cardiologists. Moreover, the low socioeconomic status and educational level of the elderly could be a possible reason for disparities in outcomes [25].

Our results also demonstrate variations in IHD-related mortality among different racial and ethnic groups in older adults, however, these differences appear less prominent when compared to younger populations [8]. In 1999, Black and NH White showed the highest mortality rates and a similar decline in AAMR over the study period when compared to other racial groups in the US. Notably, unlike the younger patients, older adults show significantly lower differences in IHD-related mortality rates among the different racial groups. Race and ethnicity could be considered a social construct, where societal, financial, political, and geographical forces influence the disparities in CVD outcomes. Despite decades of advancements in equal provision of healthcare facilities to all racial groups in the US, Black adults continue to suffer inadequate management of chronic illness, CVD being the most prominent. The recent trend indicates minimal difference in AAMR between NH White and Black, suggesting that past a specific age, the differences in socioeconomic and phenotypic status may have negligible impact on mortality outcome.

This descriptive study also underscores significant regional disparities in IHD-related mortality, with the Northeast recording the highest mortality rates, followed by the Midwest, South, and West, respectively. Multitudinous elements contribute to the regional variations in health outcomes, including the availability of top-tier healthcare services, socioeconomic conditions, lifestyle choices, and genetic predisposition of the locals [29]. Compared to the other census regions, the low incidence of IHD-related mortality in the West can largely be attributed to better access to high-quality healthcare facilities, particularly in states such as California and Washington, which ensure prompt and effective cardiovascular disease management. Socioeconomic determinants, including higher average income and education levels in the Western states, further contribute to better cardiovascular health outcomes [29]. Effective state policies in the Western region, particularly in states like California, Washington, Idaho, and Utah, have fostered healthy lifestyle choices, such as higher physical activity levels and reduced smoking rates[29–31]. These policies have led to a diminished prevalence of comorbidities, including diabetes, hypertension, obesity, and CKD, thereby enhancing overall cardiovascular health. In contrast, high population densities and relatively low socioeconomic status in the Southern and Northeastern regions have led to poor healthcare access and inimical lifestyle choices.

Additionally, the findings reveal that older adults (age ≥75 years) exhibit substantially higher IHD-related mortality rates compared to younger cohorts, including middle-aged adults, young adults, teenagers, adolescents, and children. Within the older adult cohort, the patients aged ≥85 years had a greater burden of mortality due to IHD than the patients aged between 75 and 84. This pronounced mortality in the elderly could be correlated with the age-related physiological changes in the cardiovascular system, like arterial stiffness and diminished myocardial reserve, and increased prevalence of comorbidities such as diabetes, hypertension, and CKD, which make the elderly more susceptible to IHD complications [32, 33]. The presence of several concurrent health issues can obscure or alter the symptoms of IHD in older adults, leading to delays in diagnosis and treatment. Moreover, treatment options for older adults are often constrained due to the heightened risk of post-surgical complications from invasive procedures, such as angioplasty or CABG, and increased concerns about the adverse effects and drug interactions of existing evidence-based treatments, including statins, beta-blockers, and ACE inhibitors [34, 35]. Consequently, more conservative management approaches, which might not be as effective against severe IHD, are frequently employed. Additionally, barriers to healthcare access, including mobility issues, financial constraints, and lack of medical insurance, result in delayed treatment and infrequent follow-up visits in older adults.

Analyzing the different causes of ischemic heart disease reveals that chronic IHD imposes a significantly higher mortality burden among older adults compared to acute ischemic conditions, such as myocardial infarction or angina pectoris. This disparity could be attributed to the increased prevalence of comorbidities, including diabetes, hypertension, obesity, and CKD, and the physiological age-related decline in cardiac function, which exacerbate the cumulative effect of prolonged ischemic stress on the heart. Moreover, the acute ischemic stress seen in myocardial infarction and angina pectoris is often effectively managed with contemporary interventions and evidence-based therapies, but the treatment response in chronic ischemic stress is sub-optimal. Nevertheless, there remains a pressing need for more extensive research to further elucidate the reasons behind elevated mortality rates associated with chronic IHD in older adults.

### Future directions

Based on our findings, it is important that necessary steps are taken to implement targeted public health initiatives to address IHD-related mortality, particularly in regions with high burdens. Initiatives, such as the Million Hearts program, could be expanded to rural and nonmetropolitan areas to focus on risk factor reduction, including hypertension and cholesterol management. The National Rural Health Initiative can play a pivotal role by improving access to advanced cardiovascular care, such as establishing telemedicine services and mobile clinics for early detection and management of IHD in underserved areas. Additionally, policies, such as the Healthy People 2030 framework, can guide state-level interventions to reduce disparities by focusing on equitable access to care, especially for racial and ethnic minorities who consistently face higher mortality rates. Enhancing the deployment of primary percutaneous coronary intervention (PCI) centers and incentivizing the placement of cardiovascular specialists in underserved regions through federal programs like the National Health Service Corps can address geographic disparities. These focused measures, aligned with existing initiatives, have the potential to reduce inequities and improve cardiovascular outcomes across the United States.

### Limitations

There are several limitations that should be considered. First, it is possible that the physician may be unable to appropriately diagnose the disease during the patient’s lifetime, misdiagnose the patient, or incorrectly enter the ICD codes which may lead to underestimation or misclassification of IHD as a cause of death. Additionally, the use of ’garbage codes’ in the database poses a significant challenge, as these non-specific or inaccurate codes can compromise the validity of the data and obscure true mortality trends. Second, the data available may lack information about the social determinants of available healthcare, which may influence demographic variations pertaining to IHD-related mortality. Third, there is no data on the medical treatments and interventions. Last, the database fails to provide data relevant to clinical variables including vital signs, laboratory findings, genetic analysis, and echocardiographic data that may prove helpful in understanding the phenotypic variations in IHD patients.

## Conclusion

In conclusion, the analysis has concluded that there was a gradual decline in IHD-related mortality over the course of the study. Despite reduction in the AAMRs during the last 20 years and advancements in medical therapy and technology, IHD remains a significant cause of mortality worldwide, with substantial impacts on global health and healthcare expenditures. The findings highlight important demographic and geographical disparities in IHD-related mortality rates, underscoring the need for targeted interventions and resource allocation to address areas with higher mortality rates.

## Supporting information

S1 Table Ischemic heart diseases-related deaths, stratified by sex and race, in older adults in the United States, 1999 to 2020. (DOCX)

S2 Table Ischemic heart diseases-related mortality, stratified by place of death in older adults in the United States, 1999 to 2020. (DOCX)

S3 Table Ischemic heart diseases-related age-adjusted mortality rates per 100,000, stratified in older adults in the United States, 1999 to 2020. (DOCX)

S4 Table Ischemic heart diseases-related age-adjusted mortality rates per 100,000, stratified by sex in older adults in the United States, 1999 to 2020. (DOCX)

S5 Table Ischemic heart diseases-related age-adjusted mortality rates per 100,000, stratified by race in older adults in the United States, 1999 to 2020. (DOCX)

S6 Table Ischemic heart diseases-related age-adjusted mortality rates per 100,000, stratified by age groups in older adults in the United States, 1999 to 2020. (DOCX)

S7 Table Ischemic heart diseases-related age-adjusted mortality rates per 100,000, stratified by age in the United States, 1999 to 2020. (DOCX)

S8 Table Ischemic heart diseases-related age-adjusted mortality rates per 100,000, stratified by Urban-Rural Classification in older adults in the United States, 1999 to 2020. (DOCX)

S9 Table Ischemic heart diseases-related age-adjusted mortality rates per 100,000, stratified by census regions in older adults in the United States, 1999 to 2020. (DOCX)

S10 Table Ischemic heart diseases-related age-adjusted mortality rates per 100,000, stratified by causes in older adults in the United States, 1999 to 2020. (DOCX)

S11 Table Ischemic heart diseases-related age-adjusted mortality rates per 100,000, stratified by causes in older adults in the United States, 1999 to 2020. (DOCX)
