# Supplementary material for: Demographics and regional trends of ischemic heart disease-related mortality in older adults in the United States, 1999–2020
Source: PLoS One. 2025 Jan 24;20(1):e0318073. doi: 10.1371/journal.pone.0318073 (PMC11760020; doi:10.1371/journal.pone.0318073)
Supplement: S1 Table — (DOCX) [file pone.0318073.s001.docx]

**S1 Table** Ischemic Heart Diseases-related Deaths, Stratified by Sex and Race, in Older Adults in the United States, 1999 to 2020

|  | Deaths | | | | | | | |
| --- | --- | --- | --- | --- | --- | --- | --- | --- |
| Year | **Overall** | **Women** | **Men** | **NH White** | **NH Black or African American** | **NH Asian or Pacific Islander** | **NH American Indian or Alaska Native** | **Hispanic or Latino** |
| 1999 | 443286 | 253770 | 189516 | 389490 | 32254 | 4999 | 962 | 14179 |
| 2000 | 438726 | 250014 | 188712 | 384824 | 32153 | 5123 | 996 | 14307 |
| 2001 | 432080 | 245235 | 186845 | 377260 | 32063 | 5377 | 930 | 15219 |
| 2002 | 430417 | 241596 | 188821 | 374996 | 32058 | 5729 | 1022 | 15287 |
| 2003 | 420589 | 234687 | 185902 | 365806 | 31255 | 5774 | 1109 | 15660 |
| 2004 | 397658 | 219730 | 177928 | 345282 | 29437 | 5781 | 1109 | 15299 |
| 2005 | 398588 | 218655 | 179933 | 344957 | 29401 | 5963 | 1025 | 16625 |
| 2006 | 383152 | 207965 | 175187 | 330938 | 28041 | 6199 | 1116 | 16285 |
| 2007 | 371981 | 200874 | 171107 | 320805 | 27372 | 6124 | 1094 | 16164 |
| 2008 | 370604 | 198588 | 172016 | 319534 | 26864 | 6591 | 1027 | 16004 |
| 2009 | 352129 | 185549 | 166580 | 302518 | 25580 | 6547 | 1082 | 15876 |
| 2010 | 349494 | 182510 | 166984 | 299095 | 25386 | 6729 | 1103 | 16486 |
| 2011 | 345589 | 179007 | 166582 | 295598 | 24860 | 6812 | 1179 | 16485 |
| 2012 | 338797 | 173770 | 165027 | 287998 | 24887 | 7034 | 1146 | 16769 |
| 2013 | 336249 | 170565 | 165684 | 284267 | 24750 | 7491 | 1196 | 17781 |
| 2014 | 326062 | 162773 | 163289 | 274915 | 24147 | 7307 | 1191 | 17573 |
| 2015 | 327824 | 162497 | 165327 | 274894 | 24346 | 7905 | 1285 | 18260 |
| 2016 | 320141 | 156967 | 163174 | 267083 | 24289 | 7978 | 1331 | 18562 |
| 2017 | 324430 | 156681 | 167749 | 269749 | 24483 | 8649 | 1395 | 19286 |
| 2018 | 325684 | 155465 | 170219 | 270302 | 24701 | 8947 | 1327 | 19614 |
| 2019 | 324853 | 153284 | 171569 | 268401 | 25110 | 9078 | 1292 | 20327 |
|  | 366235 | 170130 | 196105 | 295307 | 30667 | 11298 | 1665 | 26509 |
| Total | 8124568 | 4280312 | 3844256 | 6944019 | 604104 | 153435 | 25582 | 378557 |

NH, non-Hispanic.
