# Supplementary material for: Demographics and regional trends of ischemic heart disease-related mortality in older adults in the United States, 1999–2020
Source: PLoS One. 2025 Jan 24;20(1):e0318073. doi: 10.1371/journal.pone.0318073 (PMC11760020; doi:10.1371/journal.pone.0318073)
Supplement: S2 Table — (DOCX) [file pone.0318073.s002.docx]

**S2 Table** Ischemic Heart Diseases-related Mortality, Stratified by Place of Death in Older Adults in the United States, 1999 to 2020

|  | Deaths | | | |
| --- | --- | --- | --- | --- |
| Year | **Medical Facility** | **Nursing Home/Long-term Care Facility** | **Hospices** | **Home** |
| 1999 | 214615 | 134438 | Missing | 83935 |
| 2000 | 209185 | 134196 | Missing | 84096 |
| 2001 | 204010 | 131877 | Missing | 83490 |
| 2002 | 199914 | 131494 | Missing | 85336 |
| 2003 | 190273 | 128346 | 563 | 86156 |
| 2004 | 174706 | 121748 | 1048 | 84068 |
| 2005 | 171981 | 122641 | 4240 | 85450 |
| 2006 | 161541 | 117309 | 6034 | 84774 |
| 2007 | 155172 | 112405 | 7830 | 83512 |
| 2008 | 150844 | 110348 | 9384 | 83553 |
| 2009 | 137398 | 103274 | 9622 | 83119 |
| 2010 | 134436 | 102383 | 11851 | 86247 |
| 2011 | 129913 | 101287 | 13390 | 86332 |
| 2012 | 122669 | 97843 | 14960 | 88051 |
| 2013 | 118398 | 95930 | 15653 | 90663 |
| 2014 | 112633 | 92096 | 16737 | 90648 |
| 2015 | 110998 | 92193 | 18574 | 92660 |
| 2016 | 106240 | 86953 | 18771 | 94161 |
| 2017 | 105614 | 88014 | 19437 | 96594 |
| 2018 | 103969 | 86406 | 19524 | 100815 |
| 2019 | 101908 | 84204 | 20658 | 102466 |
| 2020 | 110752 | 87934 | 20386 | 128599 |
| Total | 3227169 | 2363319 | 228662 | 1984725 |
