# Supplementary material for: Demographics and regional trends of ischemic heart disease-related mortality in older adults in the United States, 1999–2020
Source: PLoS One. 2025 Jan 24;20(1):e0318073. doi: 10.1371/journal.pone.0318073 (PMC11760020; doi:10.1371/journal.pone.0318073)
Supplement: S3 Table — (DOCX) [file pone.0318073.s003.docx]

**S3 Table** Ischemic Heart Diseases-related Age-Adjusted Mortality Rates per 100,000, Stratified in Older Adults in the United States, 1999 to 2020

| Year | Age Adjusted Rate | Age Adjusted Rate  Lower 95% CI | Age Adjusted Rate  Upper 95% CI |
| --- | --- | --- | --- |
| 1999 | 2718.1 | 2710.1 | 2726.1 |
| 2000 | 2648.2 | 2640.4 | 2656.1 |
| 2001 | 2562.1 | 2554.4 | 2569.7 |
| 2002 | 2518.8 | 2511.3 | 2526.3 |
| 2003 | 2421.6 | 2414.2 | 2428.9 |
| 2004 | 2261 | 2253.9 | 2268 |
| 2005 | 2222 | 2215.1 | 2228.9 |
| 2006 | 2094.5 | 2087.8 | 2101.1 |
| 2007 | 1996.1 | 1989.6 | 2002.5 |
| 2008 | 1956.2 | 1949.9 | 1962.5 |
| 2009 | 1830 | 1823.9 | 1836 |
| 2010 | 1788.9 | 1782.9 | 1794.8 |
| 2011 | 1720.4 | 1714.6 | 1726.2 |
| 2012 | 1655.8 | 1650.1 | 1661.4 |
| 2013 | 1610.4 | 1604.9 | 1615.9 |
| 2014 | 1533.2 | 1527.9 | 1538.5 |
| 2015 | 1512 | 1506.7 | 1517.2 |
| 2016 | 1450.6 | 1445.6 | 1455.7 |
| 2017 | 1437.5 | 1432.5 | 1442.5 |
| 2018 | 1405.5 | 1400.7 | 1410.4 |
| 2019 | 1372.8 | 1368 | 1377.5 |
| 2020 | 1521.5 | 1516.5 | 1526.4 |
