# Supplementary material for: Demographics and regional trends of ischemic heart disease-related mortality in older adults in the United States, 1999–2020
Source: PLoS One. 2025 Jan 24;20(1):e0318073. doi: 10.1371/journal.pone.0318073 (PMC11760020; doi:10.1371/journal.pone.0318073)
Supplement: S4 Table — (DOCX) [file pone.0318073.s004.docx]

**S4 Table** Ischemic Heart Diseases-related Age-Adjusted Mortality Rates per 100,000, Stratified by Sex in Older Adults in the United States, 1999 to 2020

| Gender | Year | Age Adjusted Rate | Age Adjusted Rate  Lower 95% CI | Age Adjusted Rate  Upper 95% CI |
| --- | --- | --- | --- | --- |
| Female | 1999 | 2346 | 2336.8 | 2355.1 |
| Female | 2000 | 2278.6 | 2269.6 | 2287.5 |
| Female | 2001 | 2204.7 | 2196 | 2213.5 |
| Female | 2002 | 2151.5 | 2142.9 | 2160.1 |
| Female | 2003 | 2063.8 | 2055.4 | 2072.1 |
| Female | 2004 | 1916.5 | 1908.5 | 1924.6 |
| Female | 2005 | 1876.4 | 1868.5 | 1884.3 |
| Female | 2006 | 1754.5 | 1746.9 | 1762.1 |
| Female | 2007 | 1668 | 1660.7 | 1675.4 |
| Female | 2008 | 1626.2 | 1619 | 1633.4 |
| Female | 2009 | 1500.7 | 1493.8 | 1507.6 |
| Female | 2010 | 1454.7 | 1448 | 1461.5 |
| Female | 2011 | 1393.2 | 1386.7 | 1399.8 |
| Female | 2012 | 1333 | 1326.6 | 1339.4 |
| Female | 2013 | 1288.8 | 1282.6 | 1295.1 |
| Female | 2014 | 1213.2 | 1207.2 | 1219.3 |
| Female | 2015 | 1193.5 | 1187.5 | 1199.4 |
| Female | 2016 | 1138 | 1132.2 | 1143.7 |
| Female | 2017 | 1116 | 1110.4 | 1121.7 |
| Female | 2018 | 1086 | 1080.5 | 1091.5 |
| Female | 2019 | 1055.5 | 1050.1 | 1060.8 |
| Female | 2020 | 1157.6 | 1152.1 | 1163.2 |
| Male | 1999 | 3371 | 3355.6 | 3386.3 |
| Male | 2000 | 3296.9 | 3281.8 | 3311.9 |
| Male | 2001 | 3181.3 | 3166.7 | 3195.9 |
| Male | 2002 | 3156.6 | 3142.1 | 3171 |
| Male | 2003 | 3034.2 | 3020.3 | 3048.2 |
| Male | 2004 | 2847.5 | 2834.2 | 2860.9 |
| Male | 2005 | 2807.7 | 2794.7 | 2820.8 |
| Male | 2006 | 2661.4 | 2648.8 | 2673.9 |
| Male | 2007 | 2538.1 | 2526 | 2550.2 |
| Male | 2008 | 2496.9 | 2485.1 | 2508.7 |
| Male | 2009 | 2367.6 | 2356.3 | 2379 |
| Male | 2010 | 2329.3 | 2318.1 | 2340.5 |
| Male | 2011 | 2240.2 | 2229.4 | 2250.9 |
| Male | 2012 | 2165.1 | 2154.7 | 2175.6 |
| Male | 2013 | 2110.9 | 2100.8 | 2121.1 |
| Male | 2014 | 2024 | 2014.1 | 2033.8 |
| Male | 2015 | 1995.9 | 1986.3 | 2005.6 |
| Male | 2016 | 1923.1 | 1913.7 | 1932.4 |
| Male | 2017 | 1917.7 | 1908.5 | 1926.9 |
| Male | 2018 | 1879.4 | 1870.4 | 1888.3 |
| Male | 2019 | 1838.3 | 1829.6 | 1847 |
| Male | 2020 | 2051.2 | 2042.1 | 2060.3 |
