# Supplementary material for: Demographics and regional trends of ischemic heart disease-related mortality in older adults in the United States, 1999–2020
Source: PLoS One. 2025 Jan 24;20(1):e0318073. doi: 10.1371/journal.pone.0318073 (PMC11760020; doi:10.1371/journal.pone.0318073)
Supplement: S5 Table — (DOCX) [file pone.0318073.s005.docx]

**S5 Table** Ischemic Heart Diseases-related Age-Adjusted Mortality Rates per 100,000, Stratified by Race in Older Adults in the United States, 1999 to 2020

| Race | Year | Age Adjusted Rate | Age Adjusted Rate  Lower 95% CI | Age Adjusted Rate  Upper 95% CI |
| --- | --- | --- | --- | --- |
| NH American Indian or Alaska Native | 1999 | 2024.6 | 1896.3 | 2152.9 |
| NH American Indian or Alaska Native | 2000 | 1886.1 | 1768.8 | 2003.4 |
| NH American Indian or Alaska Native | 2001 | 1740.4 | 1628.3 | 1852.5 |
| NH American Indian or Alaska Native | 2002 | 1877.9 | 1762.4 | 1993.4 |
| NH American Indian or Alaska Native | 2003 | 1994.8 | 1876.9 | 2112.7 |
| NH American Indian or Alaska Native | 2004 | 1958.5 | 1842.7 | 2074.2 |
| NH American Indian or Alaska Native | 2005 | 1749 | 1641.5 | 1856.6 |
| NH American Indian or Alaska Native | 2006 | 1835.5 | 1727.4 | 1943.6 |
| NH American Indian or Alaska Native | 2007 | 1739.2 | 1635.8 | 1842.5 |
| NH American Indian or Alaska Native | 2008 | 1580.8 | 1483.8 | 1677.8 |
| NH American Indian or Alaska Native | 2009 | 1605 | 1509.2 | 1700.9 |
| NH American Indian or Alaska Native | 2010 | 1598.5 | 1504 | 1693 |
| NH American Indian or Alaska Native | 2011 | 1586.8 | 1496.1 | 1677.6 |
| NH American Indian or Alaska Native | 2012 | 1453.1 | 1368.9 | 1537.3 |
| NH American Indian or Alaska Native | 2013 | 1431.4 | 1350.3 | 1512.6 |
| NH American Indian or Alaska Native | 2014 | 1338.4 | 1262.4 | 1414.5 |
| NH American Indian or Alaska Native | 2015 | 1357.2 | 1283 | 1431.4 |
| NH American Indian or Alaska Native | 2016 | 1327.8 | 1256.4 | 1399.1 |
| NH American Indian or Alaska Native | 2017 | 1310.3 | 1241.5 | 1379.1 |
| NH American Indian or Alaska Native | 2018 | 1166.9 | 1104.1 | 1229.7 |
| NH American Indian or Alaska Native | 2019 | 1078.7 | 1019.9 | 1137.6 |
| NH American Indian or Alaska Native | 2020 | 1306.7 | 1243.9 | 1369.4 |
| NH Asian or Pacific Islander | 1999 | 1781.7 | 1731.7 | 1831.7 |
| NH Asian or Pacific Islander | 2000 | 1685.3 | 1638.7 | 1732 |
| NH Asian or Pacific Islander | 2001 | 1616.8 | 1573.1 | 1660.4 |
| NH Asian or Pacific Islander | 2002 | 1612.9 | 1570.8 | 1655 |
| NH Asian or Pacific Islander | 2003 | 1523.8 | 1484.2 | 1563.5 |
| NH Asian or Pacific Islander | 2004 | 1431.7 | 1394.6 | 1468.9 |
| NH Asian or Pacific Islander | 2005 | 1369.2 | 1334.2 | 1404.1 |
| NH Asian or Pacific Islander | 2006 | 1340.4 | 1306.9 | 1373.9 |
| NH Asian or Pacific Islander | 2007 | 1246.2 | 1214.9 | 1277.5 |
| NH Asian or Pacific Islander | 2008 | 1260.8 | 1230.3 | 1291.3 |
| NH Asian or Pacific Islander | 2009 | 1182.7 | 1154.1 | 1211.4 |
| NH Asian or Pacific Islander | 2010 | 1161.2 | 1133.5 | 1189 |
| NH Asian or Pacific Islander | 2011 | 1070.9 | 1045.4 | 1096.3 |
| NH Asian or Pacific Islander | 2012 | 1026.8 | 1002.8 | 1050.8 |
| NH Asian or Pacific Islander | 2013 | 1009.6 | 986.7 | 1032.4 |
| NH Asian or Pacific Islander | 2014 | 913 | 892.1 | 934 |
| NH Asian or Pacific Islander | 2015 | 915.8 | 895.6 | 936 |
| NH Asian or Pacific Islander | 2016 | 873.7 | 854.4 | 892.9 |
| NH Asian or Pacific Islander | 2017 | 881.1 | 862.5 | 899.7 |
| NH Asian or Pacific Islander | 2018 | 862.5 | 844.6 | 880.5 |
| NH Asian or Pacific Islander | 2019 | 826.4 | 809.3 | 843.4 |
| NH Asian or Pacific Islander | 2020 | 967.4 | 949.5 | 985.4 |
| NH Black or African American | 1999 | 2685.4 | 2656.1 | 2714.7 |
| NH Black or African American | 2000 | 2642.6 | 2613.7 | 2671.5 |
| NH Black or African American | 2001 | 2611.2 | 2582.6 | 2639.7 |
| NH Black or African American | 2002 | 2594.7 | 2566.3 | 2623.2 |
| NH Black or African American | 2003 | 2506.1 | 2478.3 | 2533.9 |
| NH Black or African American | 2004 | 2334.7 | 2308 | 2361.4 |
| NH Black or African American | 2005 | 2276.6 | 2250.6 | 2302.6 |
| NH Black or African American | 2006 | 2135.4 | 2110.4 | 2160.4 |
| NH Black or African American | 2007 | 2040.2 | 2016 | 2064.3 |
| NH Black or African American | 2008 | 1953.6 | 1930.3 | 1977 |
| NH Black or African American | 2009 | 1816.1 | 1793.8 | 1838.3 |
| NH Black or African American | 2010 | 1761.9 | 1740.2 | 1783.6 |
| NH Black or African American | 2011 | 1668.2 | 1647.5 | 1689 |
| NH Black or African American | 2012 | 1611 | 1590.9 | 1631 |
| NH Black or African American | 2013 | 1553.4 | 1534.1 | 1572.8 |
| NH Black or African American | 2014 | 1468.5 | 1449.9 | 1487.1 |
| NH Black or African American | 2015 | 1433.8 | 1415.7 | 1451.8 |
| NH Black or African American | 2016 | 1390.8 | 1373.3 | 1408.3 |
| NH Black or African American | 2017 | 1354 | 1337 | 1370.9 |
| NH Black or African American | 2018 | 1319.6 | 1303.1 | 1336 |
| NH Black or African American | 2019 | 1296.6 | 1280.6 | 1312.7 |
| NH Black or African American | 2020 | 1537.5 | 1520.3 | 1554.8 |
| NH White | 1999 | 2747.4 | 2738.8 | 2756 |
| NH White | 2000 | 2680.1 | 2671.6 | 2688.5 |
| NH White | 2001 | 2588.3 | 2580.1 | 2596.6 |
| NH White | 2002 | 2545 | 2536.8 | 2553.1 |
| NH White | 2003 | 2448.9 | 2441 | 2456.8 |
| NH White | 2004 | 2290.5 | 2282.8 | 2298.1 |
| NH White | 2005 | 2252.8 | 2245.3 | 2260.3 |
| NH White | 2006 | 2126 | 2118.7 | 2133.2 |
| NH White | 2007 | 2030.8 | 2023.7 | 2037.8 |
| NH White | 2008 | 1998.9 | 1991.9 | 2005.8 |
| NH White | 2009 | 1872.2 | 1865.5 | 1878.9 |
| NH White | 2010 | 1829.8 | 1823.2 | 1836.4 |
| NH White | 2011 | 1770.7 | 1764.3 | 1777.2 |
| NH White | 2012 | 1705.9 | 1699.6 | 1712.2 |
| NH White | 2013 | 1661.5 | 1655.3 | 1667.7 |
| NH White | 2014 | 1589.5 | 1583.5 | 1595.5 |
| NH White | 2015 | 1571 | 1565 | 1576.9 |
| NH White | 2016 | 1508.7 | 1502.9 | 1514.5 |
| NH White | 2017 | 1500.9 | 1495.1 | 1506.6 |
| NH White | 2018 | 1472 | 1466.5 | 1477.6 |
| NH White | 2019 | 1439 | 1433.5 | 1444.5 |
| NH White | 2020 | 1567.2 | 1561.6 | 1572.9 |
| Hispanic or Latino | 1999 | 2366.6 | 2327.5 | 2405.7 |
| Hispanic or Latino | 2000 | 2262.2 | 2225 | 2299.3 |
| Hispanic or Latino | 2001 | 2249.5 | 2213.6 | 2285.4 |
| Hispanic or Latino | 2002 | 2152 | 2117.7 | 2186.3 |
| Hispanic or Latino | 2003 | 2081.7 | 2048.9 | 2114.5 |
| Hispanic or Latino | 2004 | 1924.4 | 1893.7 | 1955.1 |
| Hispanic or Latino | 2005 | 1960.9 | 1930.9 | 1990.9 |
| Hispanic or Latino | 2006 | 1819.4 | 1791.3 | 1847.5 |
| Hispanic or Latino | 2007 | 1706.7 | 1680.2 | 1733.1 |
| Hispanic or Latino | 2008 | 1603.8 | 1578.9 | 1628.7 |
| Hispanic or Latino | 2009 | 1500.5 | 1477.1 | 1523.8 |
| Hispanic or Latino | 2010 | 1492.4 | 1469.5 | 1515.2 |
| Hispanic or Latino | 2011 | 1370.3 | 1349.4 | 1391.2 |
| Hispanic or Latino | 2012 | 1313.3 | 1293.4 | 1333.1 |
| Hispanic or Latino | 2013 | 1307.8 | 1288.6 | 1327.1 |
| Hispanic or Latino | 2014 | 1212.6 | 1194.7 | 1230.6 |
| Hispanic or Latino | 2015 | 1183.8 | 1166.6 | 1201 |
| Hispanic or Latino | 2016 | 1144.6 | 1128.1 | 1161.1 |
| Hispanic or Latino | 2017 | 1123.5 | 1107.6 | 1139.4 |
| Hispanic or Latino | 2018 | 1089 | 1073.7 | 1104.3 |
| Hispanic or Latino | 2019 | 1086.2 | 1071.2 | 1101.2 |
| Hispanic or Latino | 2020 | 1353.9 | 1337.6 | 1370.2 |

NH = non-Hispanic.
