# Supplementary material for: Demographics and regional trends of ischemic heart disease-related mortality in older adults in the United States, 1999–2020
Source: PLoS One. 2025 Jan 24;20(1):e0318073. doi: 10.1371/journal.pone.0318073 (PMC11760020; doi:10.1371/journal.pone.0318073)
Supplement: S6 Table — (DOCX) [file pone.0318073.s006.docx]

**S6 Table** Ischemic Heart Diseases-related Age-Adjusted Mortality Rates per 100,000, Stratified by Age in Older Adults in the United States, 1999 to 2020

| Age Group | Year | Crude Rate | Crude Rate  Lower 95% CI | Crude Rate  Upper 95% CI |
| --- | --- | --- | --- | --- |
| 75-84 years | 1999 | 1823.5 | 1816 | 1831.1 |
| 75-84 years | 2000 | 1770.2 | 1762.8 | 1777.6 |
| 75-84 years | 2001 | 1708.7 | 1701.5 | 1715.9 |
| 75-84 years | 2002 | 1664 | 1656.9 | 1671 |
| 75-84 years | 2003 | 1595.5 | 1588.6 | 1602.4 |
| 75-84 years | 2004 | 1493.9 | 1487.2 | 1500.5 |
| 75-84 years | 2005 | 1459.8 | 1453.3 | 1466.4 |
| 75-84 years | 2006 | 1380 | 1373.7 | 1386.4 |
| 75-84 years | 2007 | 1311.6 | 1305.4 | 1317.8 |
| 75-84 years | 2008 | 1279.6 | 1273.5 | 1285.7 |
| 75-84 years | 2009 | 1205.4 | 1199.5 | 1211.4 |
| 75-84 years | 2010 | 1166.2 | 1160.4 | 1172.1 |
| 75-84 years | 2011 | 1127.9 | 1122.1 | 1133.6 |
| 75-84 years | 2012 | 1080.9 | 1075.3 | 1086.5 |
| 75-84 years | 2013 | 1052.6 | 1047.1 | 1058.1 |
| 75-84 years | 2014 | 1005.9 | 1000.6 | 1011.3 |
| 75-84 years | 2015 | 988.9 | 983.6 | 994.1 |
| 75-84 years | 2016 | 949.8 | 944.8 | 954.9 |
| 75-84 years | 2017 | 936.1 | 931.1 | 941 |
| 75-84 years | 2018 | 914.7 | 910 | 919.5 |
| 75-84 years | 2019 | 894.7 | 890.1 | 899.4 |
| 75-84 years | 2020 | 999.1 | 994.2 | 1003.9 |
| 85+ years | 1999 | 5304.8 | 5282.6 | 5326.9 |
| 85+ years | 2000 | 5187 | 5165.3 | 5208.6 |
| 85+ years | 2001 | 5029.4 | 5008.2 | 5050.6 |
| 85+ years | 2002 | 4990.3 | 4969.3 | 5011.2 |
| 85+ years | 2003 | 4810 | 4789.6 | 4830.3 |
| 85+ years | 2004 | 4478.8 | 4459.4 | 4498.3 |
| 85+ years | 2005 | 4425.9 | 4406.8 | 4444.9 |
| 85+ years | 2006 | 4160.3 | 4142.2 | 4178.4 |
| 85+ years | 2007 | 3975.1 | 3957.7 | 3992.5 |
| 85+ years | 2008 | 3912.4 | 3895.4 | 3929.4 |
| 85+ years | 2009 | 3635.8 | 3619.7 | 3652 |
| 85+ years | 2010 | 3589.2 | 3573.4 | 3605.1 |
| 85+ years | 2011 | 3433.5 | 3418.4 | 3448.7 |
| 85+ years | 2012 | 3317.9 | 3303.2 | 3332.6 |
| 85+ years | 2013 | 3223.3 | 3209 | 3237.6 |
| 85+ years | 2014 | 3057.7 | 3043.9 | 3071.5 |
| 85+ years | 2015 | 3024.3 | 3010.7 | 3037.9 |
| 85+ years | 2016 | 2898.7 | 2885.5 | 2911.9 |
| 85+ years | 2017 | 2887.2 | 2874.2 | 2900.3 |
| 85+ years | 2018 | 2824.7 | 2811.9 | 2837.6 |
| 85+ years | 2019 | 2755 | 2742.3 | 2767.6 |
| 85+ years | 2020 | 3031.8 | 3018.6 | 3045.1 |
