# Supplementary material for: Demographics and regional trends of ischemic heart disease-related mortality in older adults in the United States, 1999–2020
Source: PLoS One. 2025 Jan 24;20(1):e0318073. doi: 10.1371/journal.pone.0318073 (PMC11760020; doi:10.1371/journal.pone.0318073)
Supplement: S8 Table — (DOCX) [file pone.0318073.s008.docx]

**S8 Table** Ischemic Heart Diseases-related Age-Adjusted Mortality Rates per 100,000, Stratified by Urban-Rural Classification in Older Adults in the United States, 1999 to 2020

| Metropolitan vs. non-metropolitan area | Year | Age Adjusted Rate | Age Adjusted Rate  Lower 95% CI | Age Adjusted Rate  Upper 95% CI |
| --- | --- | --- | --- | --- |
| Metropolitan | 1999 | 2717.66 | 2708.74 | 2726.59 |
| Metropolitan | 2000 | 2643.66 | 2634.93 | 2652.39 |
| Metropolitan | 2001 | 2561.93 | 2553.42 | 2570.43 |
| Metropolitan | 2002 | 2509.5 | 2501.15 | 2517.85 |
| Metropolitan | 2003 | 2409.58 | 2401.47 | 2417.68 |
| Metropolitan | 2004 | 2248.67 | 2240.89 | 2256.44 |
| Metropolitan | 2005 | 2201.22 | 2193.61 | 2208.83 |
| Metropolitan | 2006 | 2079.03 | 2071.7 | 2086.35 |
| Metropolitan | 2007 | 1975.88 | 1968.81 | 1982.95 |
| Metropolitan | 2008 | 1924.3 | 1917.38 | 1931.22 |
| Metropolitan | 2009 | 1799.61 | 1792.97 | 1806.25 |
| Metropolitan | 2010 | 1757.35 | 1750.83 | 1763.87 |
| Metropolitan | 2011 | 1689 | 1682.69 | 1695.31 |
| Metropolitan | 2012 | 1622.93 | 1616.8 | 1629.06 |
| Metropolitan | 2013 | 1575.98 | 1570 | 1581.97 |
| Metropolitan | 2014 | 1494.66 | 1488.89 | 1500.43 |
| Metropolitan | 2015 | 1471.79 | 1466.13 | 1477.46 |
| Metropolitan | 2016 | 1412.09 | 1406.6 | 1417.59 |
| Metropolitan | 2017 | 1399.34 | 1393.94 | 1404.74 |
| Metropolitan | 2018 | 1367.9 | 1362.64 | 1373.15 |
| Metropolitan | 2019 | 1332.6 | 1327.48 | 1337.73 |
| Metropolitan | 2020 | 1480.62 | 1475.28 | 1485.97 |
| Non-metropolitan | 1999 | 2723.5 | 2705.4 | 2741.6 |
| Non-metropolitan | 2000 | 2669.75 | 2651.92 | 2687.59 |
| Non-metropolitan | 2001 | 2565.18 | 2547.75 | 2582.6 |
| Non-metropolitan | 2002 | 2560.5 | 2543.13 | 2577.87 |
| Non-metropolitan | 2003 | 2474.31 | 2457.29 | 2491.33 |
| Non-metropolitan | 2004 | 2315.38 | 2298.96 | 2331.8 |
| Non-metropolitan | 2005 | 2313.69 | 2297.35 | 2330.03 |
| Non-metropolitan | 2006 | 2163.07 | 2147.37 | 2178.78 |
| Non-metropolitan | 2007 | 2086.03 | 2070.68 | 2101.38 |
| Non-metropolitan | 2008 | 2099.41 | 2084.06 | 2114.76 |
| Non-metropolitan | 2009 | 1968.01 | 1953.17 | 1982.84 |
| Non-metropolitan | 2010 | 1931.85 | 1917.23 | 1946.47 |
| Non-metropolitan | 2011 | 1864.57 | 1850.32 | 1878.81 |
| Non-metropolitan | 2012 | 1807.06 | 1793.1 | 1821.01 |
| Non-metropolitan | 2013 | 1770.38 | 1756.66 | 1784.09 |
| Non-metropolitan | 2014 | 1713.43 | 1700.01 | 1726.85 |
| Non-metropolitan | 2015 | 1700.46 | 1687.17 | 1713.74 |
| Non-metropolitan | 2016 | 1633.54 | 1620.59 | 1646.49 |
| Non-metropolitan | 2017 | 1619.61 | 1606.84 | 1632.38 |
| Non-metropolitan | 2018 | 1586.43 | 1573.94 | 1598.92 |
| Non-metropolitan | 2019 | 1567.26 | 1554.95 | 1579.57 |
| Non-metropolitan | 2020 | 1721.18 | 1708.35 | 1734.01 |
