# Supplementary material for: Demographics and regional trends of ischemic heart disease-related mortality in older adults in the United States, 1999–2020
Source: PLoS One. 2025 Jan 24;20(1):e0318073. doi: 10.1371/journal.pone.0318073 (PMC11760020; doi:10.1371/journal.pone.0318073)
Supplement: S9 Table — (DOCX) [file pone.0318073.s009.docx]

**S9 Table** Ischemic Heart Diseases-related Age-Adjusted Mortality Rates per 100,000, Stratified by States in Older Adults in the United States, 1999 to 2020

| State | Age Adjusted Rate | Age Adjusted Rate  Lower 95% CI | Age Adjusted Rate  Upper 95% CI |
| --- | --- | --- | --- |
| Alabama | 1538 | 1528.4 | 1547.5 |
| Alaska | 1276.3 | 1242.8 | 1309.9 |
| Arizona | 1596.8 | 1588.5 | 1605 |
| Arkansas | 1961.3 | 1948 | 1974.6 |
| California | 1861.1 | 1857.2 | 1865 |
| Colorado | 1435.2 | 1425.2 | 1445.2 |
| Connecticut | 1681.3 | 1670.9 | 1691.8 |
| Delaware | 2045.8 | 2021.1 | 2070.4 |
| District of Columbia | 1962.6 | 1931.3 | 1994 |
| Florida | 1792.3 | 1788 | 1796.7 |
| Georgia | 1358 | 1350.7 | 1365.3 |
| Hawaii | 1122.3 | 1108.2 | 1136.3 |
| Idaho | 1581.4 | 1563.8 | 1598.9 |
| Illinois | 1806.7 | 1800.4 | 1812.9 |
| Indiana | 1926.3 | 1917.2 | 1935.4 |
| Iowa | 2017.9 | 2005.9 | 2029.9 |
| Kansas | 1624 | 1612 | 1636 |
| Kentucky | 1965.9 | 1954.4 | 1977.4 |
| Louisiana | 1612.8 | 1602.4 | 1623.3 |
| Maine | 1782.6 | 1765.1 | 1800 |
| Maryland | 2031.8 | 2021.6 | 2042 |
| Massachusetts | 1553.5 | 1545.9 | 1561 |
| Michigan | 2179.7 | 2172.1 | 2187.3 |
| Minnesota | 1465.6 | 1457.1 | 1474.1 |
| Mississippi | 1761.4 | 1748.1 | 1774.8 |
| Missouri | 1993.8 | 1984.6 | 2003 |
| Montana | 1403 | 1384.2 | 1421.7 |
| Nebraska | 1632.2 | 1617.4 | 1646.9 |
| Nevada | 1519 | 1504.6 | 1533.4 |
| New Hampshire | 1860 | 1840.6 | 1879.3 |
| New Jersey | 2101.4 | 2093.6 | 2109.2 |
| New Mexico | 1745.8 | 1730 | 1761.6 |
| New York | 2373.5 | 2368 | 2379.1 |
| North Carolina | 1721.7 | 1714.3 | 1729.1 |
| North Dakota | 1902.1 | 1877.2 | 1927 |
| Ohio | 2158.9 | 2152.1 | 2165.8 |
| Oklahoma | 2384.3 | 2371 | 2397.6 |
| Oregon | 1505.3 | 1495.3 | 1515.4 |
| Pennsylvania | 2047.8 | 2041.9 | 2053.7 |
| Rhode Island | 2455.5 | 2432.9 | 2478.1 |
| South Carolina | 1608.3 | 1598.1 | 1618.4 |
| South Dakota | 1953.2 | 1929.9 | 1976.6 |
| Tennessee | 2186.4 | 2176.4 | 2196.5 |
| Texas | 1894.7 | 1889.4 | 1900 |
| Utah | 1258.8 | 1245 | 1272.7 |
| Vermont | 2177.2 | 2147.7 | 2206.6 |
| Virginia | 1536.7 | 1529 | 1544.5 |
| Washington | 1740.6 | 1731.9 | 1749.4 |
| West Virginia | 2412.1 | 2394.5 | 2429.6 |
| Wisconsin | 1767.7 | 1758.9 | 1776.5 |
| Wyoming | 1552.8 | 1523.6 | 1581.9 |
