# Supplementary material for: Demographics and regional trends of ischemic heart disease-related mortality in older adults in the United States, 1999–2020
Source: PLoS One. 2025 Jan 24;20(1):e0318073. doi: 10.1371/journal.pone.0318073 (PMC11760020; doi:10.1371/journal.pone.0318073)
Supplement: S10 Table — (DOCX) [file pone.0318073.s010.docx]

**S10 Table** Ischemic Heart Diseases-related Age-Adjusted Mortality Rates per 100,000, Stratified by Census Region in Older Adults in the United States, 1999 to 2020

| Census Region | Year | Age Adjusted Rate | Age Adjusted Rate Lower 95% CI | Age Adjusted Rate Upper 95% CI |
| --- | --- | --- | --- | --- |
| Northeast | 1999 | 2992 | 2974 | 3010 |
| Northeast | 2000 | 2939.1 | 2921.4 | 2956.7 |
| Northeast | 2001 | 2837.2 | 2820 | 2854.4 |
| Northeast | 2002 | 2796.6 | 2779.6 | 2813.6 |
| Northeast | 2003 | 2694.9 | 2678.4 | 2711.4 |
| Northeast | 2004 | 2537.4 | 2521.4 | 2553.4 |
| Northeast | 2005 | 2469.5 | 2453.8 | 2485.1 |
| Northeast | 2006 | 2307.3 | 2292.3 | 2322.3 |
| Northeast | 2007 | 2230.3 | 2215.6 | 2245 |
| Northeast | 2008 | 2185.1 | 2170.6 | 2199.6 |
| Northeast | 2009 | 2041.2 | 2027.3 | 2055.2 |
| Northeast | 2010 | 1965.9 | 1952.2 | 1979.6 |
| Northeast | 2011 | 1907.8 | 1894.4 | 1921.2 |
| Northeast | 2012 | 1815.6 | 1802.6 | 1828.6 |
| Northeast | 2013 | 1764.8 | 1752 | 1777.6 |
| Northeast | 2014 | 1671.9 | 1659.5 | 1684.3 |
| Northeast | 2015 | 1655.3 | 1643 | 1667.6 |
| Northeast | 2016 | 1570.9 | 1559 | 1582.9 |
| Northeast | 2017 | 1530.3 | 1518.7 | 1541.9 |
| Northeast | 2018 | 1503.1 | 1491.7 | 1514.4 |
| Northeast | 2019 | 1445.4 | 1434.4 | 1456.5 |
| Northeast | 2020 | 1619.9 | 1608.3 | 1631.6 |
| Midwest | 1999 | 2733.3 | 2717.1 | 2749.5 |
| Midwest | 2000 | 2647.8 | 2632 | 2663.6 |
| Midwest | 2001 | 2538.5 | 2523.1 | 2553.9 |
| Midwest | 2002 | 2498.8 | 2483.6 | 2514 |
| Midwest | 2003 | 2377.6 | 2362.9 | 2392.3 |
| Midwest | 2004 | 2221.9 | 2207.7 | 2236.1 |
| Midwest | 2005 | 2208 | 2193.9 | 2222 |
| Midwest | 2006 | 2108.5 | 2094.9 | 2122.1 |
| Midwest | 2007 | 2015.9 | 2002.6 | 2029.1 |
| Midwest | 2008 | 2012.1 | 1998.9 | 2025.2 |
| Midwest | 2009 | 1855.8 | 1843.2 | 1868.4 |
| Midwest | 2010 | 1818.8 | 1806.4 | 1831.3 |
| Midwest | 2011 | 1773.2 | 1761 | 1785.4 |
| Midwest | 2012 | 1718 | 1706 | 1730 |
| Midwest | 2013 | 1667.1 | 1655.4 | 1678.8 |
| Midwest | 2014 | 1604.8 | 1593.4 | 1616.3 |
| Midwest | 2015 | 1571.9 | 1560.6 | 1583.1 |
| Midwest | 2016 | 1502.9 | 1492 | 1513.9 |
| Midwest | 2017 | 1505.1 | 1494.2 | 1516 |
| Midwest | 2018 | 1472 | 1461.4 | 1482.6 |
| Midwest | 2019 | 1442.1 | 1431.6 | 1452.5 |
| Midwest | 2020 | 1615.4 | 1604.4 | 1626.4 |
| South | 1999 | 2640.7 | 2627.2 | 2654.2 |
| South | 2000 | 2591.2 | 2578 | 2604.5 |
| South | 2001 | 2518 | 2505.1 | 2531 |
| South | 2002 | 2471.6 | 2458.8 | 2484.4 |
| South | 2003 | 2373 | 2360.6 | 2385.5 |
| South | 2004 | 2206.6 | 2194.7 | 2218.5 |
| South | 2005 | 2180.4 | 2168.7 | 2192.1 |
| South | 2006 | 2029.1 | 2017.9 | 2040.2 |
| South | 2007 | 1932 | 1921.2 | 1942.7 |
| South | 2008 | 1878.7 | 1868.2 | 1889.2 |
| South | 2009 | 1780.2 | 1770 | 1790.3 |
| South | 2010 | 1753.2 | 1743.2 | 1763.2 |
| South | 2011 | 1650.2 | 1640.7 | 1659.8 |
| South | 2012 | 1603.3 | 1594 | 1612.6 |
| South | 2013 | 1560 | 1551 | 1569.1 |
| South | 2014 | 1492.3 | 1483.6 | 1501.1 |
| South | 2015 | 1465.7 | 1457.1 | 1474.2 |
| South | 2016 | 1409.9 | 1401.6 | 1418.2 |
| South | 2017 | 1402.2 | 1394 | 1410.3 |
| South | 2018 | 1377.6 | 1369.6 | 1385.5 |
| South | 2019 | 1356.6 | 1348.9 | 1364.4 |
| South | 2020 | 1509.6 | 1501.5 | 1517.7 |
| West | 1999 | 2526.5 | 2509 | 2544.1 |
| West | 2000 | 2422.2 | 2405.2 | 2439.3 |
| West | 2001 | 2361.9 | 2345.3 | 2378.4 |
| West | 2002 | 2315.1 | 2298.8 | 2331.3 |
| West | 2003 | 2256.4 | 2240.6 | 2272.2 |
| West | 2004 | 2098.2 | 2083.1 | 2113.4 |
| West | 2005 | 2040.4 | 2025.7 | 2055.1 |
| West | 2006 | 1958.9 | 1944.7 | 1973.2 |
| West | 2007 | 1831.9 | 1818.3 | 1845.5 |
| West | 2008 | 1781.7 | 1768.4 | 1794.9 |
| West | 2009 | 1665.3 | 1652.6 | 1678 |
| West | 2010 | 1630.5 | 1618.1 | 1643 |
| West | 2011 | 1588.9 | 1576.9 | 1601 |
| West | 2012 | 1515 | 1503.3 | 1526.6 |
| West | 2013 | 1480.1 | 1468.7 | 1491.5 |
| West | 2014 | 1390 | 1379.1 | 1400.9 |
| West | 2015 | 1387.9 | 1377.2 | 1398.7 |
| West | 2016 | 1350.6 | 1340.1 | 1361 |
| West | 2017 | 1336 | 1325.7 | 1346.3 |
| West | 2018 | 1290.8 | 1280.9 | 1300.8 |
| West | 2019 | 1260.7 | 1251 | 1270.4 |
| West | 2020 | 1358.1 | 1348.2 | 1368 |
