# Supplementary material for: Demographics and regional trends of ischemic heart disease-related mortality in older adults in the United States, 1999–2020
Source: PLoS One. 2025 Jan 24;20(1):e0318073. doi: 10.1371/journal.pone.0318073 (PMC11760020; doi:10.1371/journal.pone.0318073)
Supplement: S11 Table — (DOCX) [file pone.0318073.s011.docx]

**S11 Table** Ischemic Heart Diseases-related Age-Adjusted Mortality Rates per 100,000, Stratified by Causes in Older Adults in the United States, 1999 to 2020

| Census Region | Year | Age Adjusted Rate | Age Adjusted Rate Lower 95% CI | Age Adjusted Rate Upper 95% CI |
| --- | --- | --- | --- | --- |
| Angina Pectoris | 1999 | 13 | 12.5 | 13.6 |
| Angina Pectoris | 2000 | 12.9 | 12.3 | 13.4 |
| Angina Pectoris | 2001 | 10.7 | 10.2 | 11.2 |
| Angina Pectoris | 2002 | 9.4 | 8.9 | 9.8 |
| Angina Pectoris | 2003 | 7.8 | 7.4 | 8.2 |
| Angina Pectoris | 2004 | 6.4 | 6 | 6.8 |
| Angina Pectoris | 2005 | 5.8 | 5.4 | 6.1 |
| Angina Pectoris | 2006 | 4.7 | 4.4 | 5 |
| Angina Pectoris | 2007 | 4.3 | 4 | 4.6 |
| Angina Pectoris | 2008 | 3.8 | 3.6 | 4.1 |
| Angina Pectoris | 2009 | 3.5 | 3.2 | 3.7 |
| Angina Pectoris | 2010 | 3.4 | 3.2 | 3.7 |
| Angina Pectoris | 2011 | 3 | 2.8 | 3.2 |
| Angina Pectoris | 2012 | 3.1 | 2.9 | 3.4 |
| Angina Pectoris | 2013 | 3.1 | 2.9 | 3.3 |
| Angina Pectoris | 2014 | 2.9 | 2.7 | 3.2 |
| Angina Pectoris | 2015 | 3.1 | 2.9 | 3.4 |
| Angina Pectoris | 2016 | 4.6 | 4.3 | 4.8 |
| Angina Pectoris | 2017 | 5 | 4.7 | 5.3 |
| Angina Pectoris | 2018 | 5.4 | 5.1 | 5.7 |
| Angina Pectoris | 2019 | 5.6 | 5.3 | 5.9 |
| Angina Pectoris | 2020 | 6.7 | 6.4 | 7 |
| Chronic Ischemic Heart Disease | 1999 | 2217.1 | 2209.9 | 2224.4 |
| Chronic Ischemic Heart Disease | 2000 | 2152.5 | 2145.4 | 2159.6 |
| Chronic Ischemic Heart Disease | 2001 | 2084.7 | 2077.9 | 2091.6 |
| Chronic Ischemic Heart Disease | 2002 | 2052 | 2045.2 | 2058.8 |
| Chronic Ischemic Heart Disease | 2003 | 1979.6 | 1973 | 1986.2 |
| Chronic Ischemic Heart Disease | 2004 | 1860.3 | 1854 | 1866.7 |
| Chronic Ischemic Heart Disease | 2005 | 1833.2 | 1827 | 1839.5 |
| Chronic Ischemic Heart Disease | 2006 | 1732.5 | 1726.5 | 1738.6 |
| Chronic Ischemic Heart Disease | 2007 | 1657.2 | 1651.3 | 1663 |
| Chronic Ischemic Heart Disease | 2008 | 1616.2 | 1610.5 | 1622 |
| Chronic Ischemic Heart Disease | 2009 | 1518.4 | 1512.9 | 1524 |
| Chronic Ischemic Heart Disease | 2010 | 1488.3 | 1482.9 | 1493.8 |
| Chronic Ischemic Heart Disease | 2011 | 1431.7 | 1426.4 | 1437 |
| Chronic Ischemic Heart Disease | 2012 | 1380.6 | 1375.4 | 1385.7 |
| Chronic Ischemic Heart Disease | 2013 | 1343.6 | 1338.6 | 1348.6 |
| Chronic Ischemic Heart Disease | 2014 | 1280.6 | 1275.8 | 1285.5 |
| Chronic Ischemic Heart Disease | 2015 | 1261.7 | 1257 | 1266.5 |
| Chronic Ischemic Heart Disease | 2016 | 1212.7 | 1208.1 | 1217.4 |
| Chronic Ischemic Heart Disease | 2017 | 1206 | 1201.5 | 1210.6 |
| Chronic Ischemic Heart Disease | 2018 | 1182.2 | 1177.7 | 1186.6 |
| Chronic Ischemic Heart Disease | 2019 | 1163.8 | 1159.5 | 1168.2 |
| Chronic Ischemic Heart Disease | 2020 | 1297.7 | 1293.1 | 1302.2 |
| Myocardial Infarction | 1999 | 877 | 872.5 | 881.5 |
| Myocardial Infarction | 2000 | 853.8 | 849.3 | 858.2 |
| Myocardial Infarction | 2001 | 814.4 | 810.1 | 818.7 |
| Myocardial Infarction | 2002 | 787.1 | 782.9 | 791.3 |
| Myocardial Infarction | 2003 | 739.3 | 735.3 | 743.4 |
| Myocardial Infarction | 2004 | 669.2 | 665.4 | 673 |
| Myocardial Infarction | 2005 | 639 | 635.3 | 642.7 |
| Myocardial Infarction | 2006 | 587.5 | 584 | 591 |
| Myocardial Infarction | 2007 | 547.3 | 544 | 550.7 |
| Myocardial Infarction | 2008 | 538 | 534.7 | 541.3 |
| Myocardial Infarction | 2009 | 488.7 | 485.6 | 491.9 |
| Myocardial Infarction | 2010 | 468.8 | 465.7 | 471.8 |
| Myocardial Infarction | 2011 | 446.7 | 443.8 | 449.7 |
| Myocardial Infarction | 2012 | 426.6 | 423.7 | 429.4 |
| Myocardial Infarction | 2013 | 410.7 | 407.9 | 413.5 |
| Myocardial Infarction | 2014 | 387.1 | 384.4 | 389.8 |
| Myocardial Infarction | 2015 | 379.7 | 377.1 | 382.3 |
| Myocardial Infarction | 2016 | 361.1 | 358.5 | 363.6 |
| Myocardial Infarction | 2017 | 348.9 | 346.4 | 351.4 |
| Myocardial Infarction | 2018 | 336.1 | 333.7 | 338.4 |
| Myocardial Infarction | 2019 | 319 | 316.7 | 321.3 |
| Myocardial Infarction | 2020 | 336.3 | 334 | 338.6 |
| Other Ischemic Heart Diseases | 1999 | 25.5 | 24.8 | 26.3 |
| Other Ischemic Heart Diseases | 2000 | 25.1 | 24.4 | 25.9 |
| Other Ischemic Heart Diseases | 2001 | 24.4 | 23.6 | 25.1 |
| Other Ischemic Heart Diseases | 2002 | 23.8 | 23.1 | 24.5 |
| Other Ischemic Heart Diseases | 2003 | 21.8 | 21.1 | 22.5 |
| Other Ischemic Heart Diseases | 2004 | 18.5 | 17.9 | 19.1 |
| Other Ischemic Heart Diseases | 2005 | 25.6 | 24.9 | 26.4 |
| Other Ischemic Heart Diseases | 2006 | 27.6 | 26.8 | 28.3 |
| Other Ischemic Heart Diseases | 2007 | 26.2 | 25.5 | 27 |
| Other Ischemic Heart Diseases | 2008 | 27 | 26.3 | 27.8 |
| Other Ischemic Heart Diseases | 2009 | 25.8 | 25 | 26.5 |
| Other Ischemic Heart Diseases | 2010 | 26.6 | 25.9 | 27.3 |
| Other Ischemic Heart Diseases | 2011 | 25.5 | 24.8 | 26.2 |
| Other Ischemic Heart Diseases | 2012 | 24.2 | 23.5 | 24.9 |
| Other Ischemic Heart Diseases | 2013 | 24.6 | 24 | 25.3 |
| Other Ischemic Heart Diseases | 2014 | 24.2 | 23.6 | 24.9 |
| Other Ischemic Heart Diseases | 2015 | 24 | 23.3 | 24.6 |
| Other Ischemic Heart Diseases | 2016 | 22 | 21.4 | 22.7 |
| Other Ischemic Heart Diseases | 2017 | 22.2 | 21.6 | 22.8 |
| Other Ischemic Heart Diseases | 2018 | 21.8 | 21.2 | 22.4 |
| Other Ischemic Heart Diseases | 2019 | 21.7 | 21.1 | 22.3 |
| Other Ischemic Heart Diseases | 2020 | 22.4 | 21.8 | 23 |
